# Advancing Breast Cancer Therapeutics: Targeted Gene Delivery Systems Unveiling the Potential of Estrogen Receptor-Targeting Ligands

**DOI:** 10.34133/bmr.0087

**Published:** 2024-09-24

**Authors:** Jung Ro Lee, Young-Min Kim, Eun-Ji Kim, Mi-Kyeong Jang, Seong-Cheol Park

**Affiliations:** ^1^ National Institute of Ecology (NIE), Seocheon 33657, Korea.; ^2^ Department of Chemical Engineering, College of Engineering, Sunchon National University, Suncheon, Jeonnam 57922, Korea.

## Abstract

Although curcumin has been well known as a phytochemical drug that inhibits tumor promotion by modulating multiple molecular targets, its potential was not reported as a targeting ligand in the field of drug delivery system. Here, we aimed to assess the tumor-targeting efficiency of curcumin and its derivatives such as phenylalanine, cinnamic acid, coumaric acid, and ferulic acid. Curcumin exhibited a high affinity for estrogen receptors through a pull-down assay using the membrane proteins of MCF-7, a breast cancer cell line, followed by designation of a polymer-based gene therapy system. As a basic backbone for gene binding, dextran grafted with branched polyethylenimine was synthesized, and curcumin and its derivatives were linked to lysine dendrimers. In vitro and in vivo antitumor effects were evaluated using plasmid DNA expressing anti-*bcl-2* short hairpin RNA. All synthesized gene carriers showed excellent DNA binding, protective effects against nuclease, and gene transfection efficiency in MCF-7 and SKBr3 breast cancer cells. Preincubation with curcumin or 17α-estradiol resulted in a marked dose-dependent decrease in gene transfer efficiency and suggested targeting specificity of curcumin. Our study indicates the potential of curcumin and its derivatives as novel targeting ligands for tumor cells and tissues.

## Introduction

Cancer continues to be one of the most lethal diseases worldwide, despite the increasing number of interventions available. Advances in cancer diagnosis and treatment have not improved overall survival rates for various reasons, including drug resistance, metastasis, and side effects [1]. Among current antitumor treatments, chemotherapy plays an essential role, but most chemical agents have low specificity for cancer cells, resulting in toxicity and side effects in the body [2]. To overcome these obstacles, many studies have focused on tumor-specific targeting systems, which are expected to play an important role in the next generation of chemotherapy treatments [3,4].

Drug delivery systems are classified into passive delivery systems, which transport drugs by taking advantage of anatomical differences between normal and tumor tissues, and active delivery systems, which deliver drugs intensively to specific tissues through targeting ligands [2,5–7]. Unlike treatment via passive drug delivery, nanoparticles with a targeting moiety can effectively accumulate in cancer tissues and cells by binding to receptors expressed in specific tumor tissues and are efficiently introduced into cells through receptor-mediated endocytosis. Therefore, cancer treatments using targeting moieties can be applied to various cancer cells using 1 effective drug via the selection of targeting ligands that can bind to receptors overexpressed in specific tumor tissues [8,9].

Gene therapies are being developed for various diseases, with the development of effective gene delivery systems a primary area of research. Recently, nonviral vectors, including cationic polymers, lipids, dendrimers, and peptides, have been proposed as safer alternatives to gene therapy and offer several advantages, such as mild immune responses, ability to produce the vectors in large quantities with high reproducibility, acceptable cost, stability to storage in nonexotic conditions, and ease of administration to patients [10–12]. Nonviral gene delivery systems are typically composed of genes condensed into nanoparticles prepared with cationic polymers such as polylysine, polyethyleneimine (PEI), polyamidoamine, chitosan, and dendrimers [13,14]. PEI is a positively charged cationic polymer with various structures such as branched or linear polymer chains that undergoes functionalization for conjugation [15]. Lysine dendrimers can strongly bind to genes via electrostatic interactions. They are expected to exhibit high gene transfection efficiency because of their rapid endosomal escape via the proton sponge mechanism in a broad range of cell types when compared with the efficiency of other cationic polymers.

Dextran is a biodegradable, biocompatible, and nonimmunogenic polysaccharide that can be used as a potential module for delivery systems. It inhibits nonspecific interactions with serum proteins and prolongs the biological half-life of its conjugates, thereby increasing their stability in vivo. Dextran has been shown to improve cell viability and serum stability of PEI-based systems, with promising results in gene delivery applications [16–18].

Curcumin demonstrates a wide range of pharmacological activities, including anti-inflammatory, anticancer, antioxidant, wound-healing, and antimicrobial effects [19–21]. It suppresses the proliferation of various tumor cells, including those from colorectal cancer, pancreatic cancer, breast cancer, prostate cancer, multiple myeloma, lung cancer, and oral cancer [19]. Curcumin inhibits cancer promotion by modulating multiple molecular targets such as transcription factors, enzymes, cell cycle proteins, cell surface adhesion proteins, components of cell survival pathways, and cytokines [22]. Despite its therapeutic efficacy, curcumin has some disadvantages, including poor aqueous solubility, relatively low bioavailability, and intense staining. To overcome these limitations, various curcumin formulations, including curcumin in nanoparticles, liposomes, micelles, and phospholipid complexes, have been designed [23,24]. Curcumin has only been studied for use in nanoparticles as a therapeutic drug, but its potential for other uses has not been explored.

This study aimed to assess the tumor-targeting efficiency of 5 targeting moieties: phenylalanine, cinnamic acid, coumaric acid, ferulic acid, and curcumin. Here, we hypothesized that curcumin could best be used as a tumor-targeting ligand if a specific membrane receptor that transports it was identified. We selected MCF-7, a breast cancer cell line, because curcumin has shown excellent antitumor activity in breast cancer cells, along with diverse other cancer cell types. The targeting capacities of polyplexes complexed with PEI-grafted dextran, lysine-dendrimer-linked targeting ligands, and short hairpin RNA (shRNA)-expressing plasmid DNA (pDNA) were investigated using transfection efficiency, confocal microscopy, and competition assays. The in vitro and in vivo antitumor effects of these formulations were evaluated using RNA-silencing gene delivery. Our findings will help confirm the potential of curcumin and its derivatives as novel targeting ligands for tumor cells and tissues.

## Materials and Methods

### Reagents

Dextran (*Leuconostoc* spp. molecular weight [Mw] 6,000 Da), branched PEI (bPEI Mw 2,000 Da), N-hydroxysuccinimide (NHS), 1-ethyl-3-(3-dimethylaminopropyl) carbodiimide hydrochloride, 1,1′-carbonyldiimidazole (CDI), N,N'-dicyclohexylcarbodiimide (DCC), 4-dimethylaminopyridine, trans-cinnamic acid, p-coumaric acid, trans-ferulic acid, curcumin, α-estradiol, and heparin were purchased from Sigma-Aldrich Inc. (St. Louis, MO, USA). Fluorenylmethyloxycarbonyl (Fmoc)-Glu(OtBu)-OH, Fmoc-Phe-OH, Fmoc-Lys(Boc)-OH, Fomc-Cys(Trt)-OH, and ethyl 2-cyano-2-(hydroxyimino)acetate (Oxyma) were purchased from CEM Co. (Matthews, NC, USA). Fmoc-Lys(Fomc)-OH and *N,N′*-diisopropylcarbodiimide were purchased from TCI Co. (Tokyo, Japan). Maleimide-polyethylene glycol-amine (Mw 3,400 Da) (Mal-PEG-NH_2_) was obtained from BiochemPeg Scientific Inc. (Watertown, MA, USA). DNase Kits were purchased from Promega (Madison, WI, USA). TurboFect, ActinGreen 488, LysoTracker Green DND-26, and ProLong Gold Antifade Mountant were obtained from Thermo Fisher Scientific Co. (Seoul, Korea). Flamma 675 NHS ester was purchased from BioActs (Incheon, Korea). Dialysis tubes (Spectra Por-6, Mw cutoff = 3,500 g/mol) were purchased from SpectraPor Laboratories, Inc. (Rancho Dominguez, CA, USA). Cell Counting Kit-8 (CCK-8) was purchased from Dojindo Laboratories Co. (Kamimashiki-gun, Kumamoto, Japan). Dulbecco’s modified Eagle’s medium, Roswell Park Memorial Institute (RPMI) 1640 medium, and fetal bovine serum (FBS) were supplied by Thermo Fisher Scientific Inc. (Waltham, MA, USA). pEGFP-N1 DNA was obtained from BD Biosciences Inc. (Franklin Lakes, NJ, USA). The psiRNA-hBCL2 and plasmocin were purchased from InvivoGen (San Diego, CA, USA). All other solvents and chemicals were of reagent grade and used as received.

### Cell culture

Human prostate cancer cells (PC3), breast cancer cells (SKBr-3, MDA-MB-231, and MCF-7), ovarian cancer cells (SKOV-3), and colon carcinoma cells (DLD-1) were obtained from the Korean Cell Line Bank (Seoul, Korea). The cells were cultured in RPMI 1640 medium supplemented with 10% FBS and antibiotics (100 U/ml penicillin, 100 μg/ml streptomycin, and 10 μg/ml plasmocin). All cells were grown at 37 °C in a humidified atmosphere containing 5% CO_2_.

### Peptide synthesis

Peptides were prepared using solid-phase methods with Fmoc-protected amino acids on a Liberty Microwave Peptide Synthesizer (CEM Co.). Fmoc-Lys(Fmoc)-OH was used as the backbone material for dendrimer peptide synthesis. Fully protected K_8_ dendrimers (K_8_[K_2_K]_2_KC) (dK) were synthesized on 2-chlorotrityl chloride resin (ChemPep, Inc., Wellington, FL, USA) with Oxyma/*N,N′*-diisopropylcarbodiimide (coupling agent) and 20% piperidine (v/v, deprotection agent) in dimethylformamide (DMF) under 90 °C heating. For each coupling step, Fmoc-protected amino acids and coupling agents were added in 5-fold molar excess to the resin concentration. After cleavage from the resin in the presence of a cocktail solution (trifluoroacetic acid [TFA]/triisopropylsilane/diH_2_O (95: 2.5: 2.5, v/v/v) for 50 min at 40 °C, the fully protected peptide was precipitated and freeze-dried.

### Preparation of the copolymer and targeting ligands

To prepare dextran copolymer conjugated with bPEI (DP), carboxyl group of Fmoc-Glu (OtBu)-OH (E) was activated with 1-ethyl-3-(3-dimethylaminopropyl) carbodiimide hydrochloride in a cosolvent of 50 mM 2-(*N*-morpholino)ethanesulfonic acid (MES) buffer (pH 4.95) and dimethyl sulfoxide (DMSO) at 25 °C for 30 min. The bPEI was added drop-wise to activated E (molar ratio, 1:1) and incubated at 25 °C for 6 h (EP). After acetone precipitation, OtBu protecting groups on EP were cleaved with 95% TFA, and the EP was reprecipitated using ethyl ether. The product was dialyzed to remove unreacted Fmoc-amino acids and cleaved OtBu with DMSO for 1 d and further dialyzed in deionized water (DW) for 2 d, followed by freeze drying. Next, EP was conjugated chemically to dextran in a 1% molar ratio with DCC/4-dimethylaminopyridine in DMSO at 25 °C for 12 h, resulting in DP. Then, DP was precipitated with acetone, and Fmoc groups were deprotected with 20% piperidine/DMF at 50 °C for 2 h. DP was dialyzed with DW for 48 h and freeze-dried (Fig. S1).

To synthesize targeting ligands, carboxyl group of Fmoc-Phe-OH (Ph), trans-cinnamic acid (Ci), p-coumaric acid (Co), and trans-ferulic acid (Fe) were activated with DCC/NHS in DMSO at 25 °C for 15 min, followed by incubation with the Mal-PEG-NH_2_ solution (molar ratio, 1:1) at 25 °C for 12 h. Fmoc of Ph-PEG-Mal was removed by incubation with 20% piperidine/DMF at 50 °C for 2 h. Hydroxyl groups of curcumin were activated with CDI in DMSO at 60 °C for 15 min. The activated dextran was then added to the Mal-PEG-NH_2_ solution (molar ratio, 1:1) and stirred overnight at 25 °C. All reactants were first dialyzed in DMSO for 24 h to remove unreacted CDI, Ph, Ci, Co, Fe, and Cu. After dialyzing with DW for 48 h, the solution was freeze-dried. The PEGylated ligands were reacted with K_8_ dendrimer (dK) (K_8_[K_2_K]_2_KC) (molar ratio, 1:1) in 50 mM 4-(2-hydrozyethyl)-1-piperazinnethanesulfonic acid buffer (pH 8.0) buffer at 25 °C for 4 h via a facile maleimide-thiol “click” reaction. The solution was dialyzed against DW for 24 h and then freeze-dried (Fig. S2). All materials from these synthesis steps were characterized by ^1^H-nuclear magnetic resonance (NMR, AVANCE 400FT NMR 400 MHz, Bruker) spectroscopy (Figs. S3 to S7).

### Pull-down assay and western blot analysis

MCF-7 cells were grown at 37 °C in a 5% CO_2_ incubator. Cytosolic and membrane proteins were isolated using a Mem-PER plus membrane protein extraction kit (Thermo Fisher Scientific Inc.) according to the manufacturer’s protocol. Cells were collected by centrifugation at 300 × *g* for 5 min, and the cell pellets were washed twice with a cell wash and centrifuged again. The supernatant was carefully discarded and permeabilization buffer with a protease and phosphate inhibitor cocktail (Thermo Fisher Scientific Inc.) added. Then, the cell pellet was incubated at 4 °C for 10 min with constant mixing, followed by centrifugation for 15 min at 16,000 × *g*. The supernatant containing cytosolic proteins was transferred to a new tube, and the residual cell pellet was resuspended in a solubilization buffer with a protease and phosphate inhibitor cocktail and incubated at 4 °C for 30 min with constant mixing. The tubes were then centrifuged at 16,000 × *g* for 15 min at 4 °C to collect the supernatant containing solubilized membrane proteins [25,26]. These membrane proteins were stored at −80 °C for future use.

For pull-down assay, membrane proteins were dialyzed with phosphate-buffered saline (PBS) (10 mM Na_2_HPO_4_, 1.8 mM KH_2_PO_4_, 2.7 mM KCl, and 137 mM NaCl, pH 7.4) for 3 h at 4 °C [27]. The curcumin Sepharose 4B resin was washed 3 times with cold PBS. The mixture was centrifuged at 2,000 × *g* for 5 min, the supernatant was discarded, and then 600 μl of PBS was added to the Sepharose resins to prepare a 50% slurry. Following this, 3 mg of membrane proteins was combined with the curcumin Sepharose resins and incubated overnight at 4 °C on a rotary shaker. These resins were washed thrice with a 10-bed volume of cooled PBS and centrifuged at 2,000 × *g* for 5 min at 4 °C.

Membrane proteins subjected to pull-down assays were analyzed by western blotting. Membrane proteins were separated using 12% sodium dodecyl sulfate polyacrylamide gel electrophoresis (SDS-PAGE) (Bio-Rad) and transferred onto polyvinylidene fluoride membranes (Bio-Rad), and then the blots were blocked with 5% skim milk (Becton Dickinson and Company, Franklin Lakes, NJ, USA). Antibody used for detection included human estrogen receptor α (ERα) (8644S, Cell Signaling Technology, Danvers, MA, USA; 1:1,000) [28]. The secondary antibody used was an anti-rabbit antibody (7074S; Cell Signaling Technology, Danvers, MA, USA; 1:10,000). The blots were incubated with ECL reagent (Thermo Fisher Scientific Inc.) and visualized using a ChemiDoc XRS+ Imaging System (Bio-Rad). Western blot images were analyzed quantitatively using Image Lab software (Bio-Rad).

### Molecular docking

The structures of ERα (7qvj and 8duk) and Erβ (4J26 and 5toa) were obtained from RCSB Protein Data Bank (RCSB PDB). The structure of curcumin (CC9) was obtained from National Center for Biotechnology Information structure. These structures were prepared for simulating docking in a Patch Dock server (https://bioinfo3d.cs.tau.ac.il/PatchDock/) and visualized using PyMOL software.

### Electrophoretic mobility shift assay and deoxyribonuclease stability

The pEGFP-N1 amplified in *Escherichia coli* DH5α cells were purified using a pDNA midiprep kit (GeneJET Plasmid Midiprep Kit, Thermo Fisher Scientific Inc.) according to the manufacturer’s protocol. The purity of pDNA was assessed by measuring the absorbance ratio at optical density at 260 nm (OD_260_)/OD_280_. DP polymer was mixed with dKPh (DPKPh), dKCi (DPKCi), dKCo (DPKCo), dKFe (DPKFe), or dKCu (DPKCu) at 9.5:0.5, 9:1, or 8.2 weight ratio in PBS solution, after which the polymers were complexed with pDNA at various weight ratios ranging from 0.01 to 64 in PBS and incubated for 30 min at 25 °C. DNA protection capacity of the polymers was assessed by adding deoxyribonuclease (DNase) after polyplex formation. Briefly, 1.5 μl of RNase-Free DNase I (1 U/μl in buffer containing 100 mM tris, 25 mM MgCl_2_, and 5 mM CaCl_2_) was incubated with polyplexes for 20 min at 37 °C, followed by inactivation of DNase by incubation at 75 °C for 10 min. DNA was released from polyplexes by treatment with heparin (60 units) for 10 min at 25 °C. All complexes were electrophoresed on a 0.8% (w/v) agarose gel at 100 V, stained with SafeView classic (Applied Biological Materials Inc., Richmond, BC, Canada), and illuminated using a blue light-emitting diode illuminator.

### Size distribution and zeta potential analysis of polyplexes

The particle sizes and zeta potentials of DP, DPK, DPKPh, DPKCi, DPKCo, DPKFe, and DPKCu complexed with pDNA were investigated using an ELS-8000 electrophoretic light-scattering spectrophotometer (Otsuka Electronics, Osaka, Japan) equipped with a He-Ne laser operating at 25 °C and a fixed scattering angle of 90°.

### Transfection efficiency of polyplexes in various cell lines

The transfection efficiencies of DPK, DPKPh, DPKCi, DPKCo, DPKFe, and DPKCu polyplexes with pEGFP-N1 were investigated in PC3, SKBr-3, MDA-MB-231, MCF-7, SKOV-3, DLD-1, and HaCaT cells. Cells were seeded at 2 × 10^4^ cells/well in 24-well plates and incubated for 24 h. The medium was aspirated, and polyplexes were added to wells at indicated weight/weight ratios in antibiotic-free medium supplemented 10% FBS. TurboFect was used as a control transfection reagent. After incubation for 48 h, green fluorescent protein (GFP) expression in the cells was observed under a fluorescence microscope (IX71, Olympus, Japan). In addition, quantitative analysis of transfected cells was performed using an Attune NxT acoustic focusing cytometer (Thermo Fisher Scientific Co.).

### Cellular uptake and localization of nanoparticles

Cellular uptake of the polyplexes was determined by confocal laser scanning microscopy. The DP polymers were labeled with NHS-activated Flamma 675 dye at a molar ratio of 1:1 in PBS. Flamma 675-labeled DP/DP/DPK, DPKPh, DPKCi, DPKCo, DPKFe, and DPKCu polymers were complexed with pET28(a) at a 9:1 weight ratio and added to wells, where MCF-7 cells were seeded at a density of 2×10^5^ cells/ml in slide glass culture wells (SPL Life Science, Pocheon-si, Gyeonggi-do, Korea) and incubated for 12 h. After incubation for 2 h, the glass slides were rinsed with PBS, stained with ActinGreen 488, and mounted with an antifade mountant with 4′,6-diamidino-2-phenylindole (DAPI) according to the manufacturer’s protocols. To investigate cellular localization and endosomal escape of polyplexes in MCF-7 cells, the polyplexes were prepared by the same method of cellular uptake. After incubating the polyplexes for 6 h, the glass slides were washed with PBS. Then, the cells were stained with LysoTracker and mounted using an antifade mountant with DAPI according to the manufacturer’s protocols. The intracellular polyplexes were observed using a Zeiss LSM-510 confocal microscope (ZEISS, Jena, Germany) under identical conditions. The images were recorded digitally in a 512 × 512-pixel format.

### Cytotoxicity and antitumor activity

Hemolytic and cytotoxic effects of the polyplexes were evaluated using rat erythrocytes and HaCaT cells, respectively. Fresh blood obtained from a healthy rat tail was transferred to a heparin tube, and rat red blood cells (rRBCs) were collected by washing with PBS until the supernatant was clear. Polymers mixed with dKs at 9:1 (w/w) were complexed with psiRNA-hBCL2 at 2:1 (w/w) and then added to 8% rRBCs (v/v) in PBS, followed by incubation with mild agitation for 1 h at 37 °C. The cells were centrifuged for 10 min at 800 × *g*, and the absorbance of the supernatants was measured at 414 nm. The controls for zero (blank) and 100% hemolysis were PBS alone and 0.3% (v/v) Triton X-100, respectively. Each measurement was conducted in triplicate. Cytotoxic effects and antitumor activities of the polyplexes were evaluated by CCK-8 assay. A total of 5 × 10^3^ cells/well were seeded in a 96-well plate and incubated for 24 h. Subsequently, the polymers mixed with dKs at 9:1 (w/w) were complexed with psiRNA-hBCL2 at 2:1 (w/w) in a minimal volume of Dulbecco’s PBS. The polyplex solutions added to the culture medium (8× the complex volume) were placed in cell plates, after which plates were incubated for 24 or 48 h at 37 °C. A 10-μl CCK-8 solution was added to each well, and the cells were further incubated for 4 h at 37 °C. Absorbances were measured at 430 nm (optical density) and 490 nm (with background subtracted) using a microplate reader (SpectraMax M5 Microplate Reader, Molecular Devices, Sunnyvale, CA, USA). Then, 100% cytotoxicity was determined following treatment with 0.3% (v/v) Triton X-100.

### Titration assay in gene transfection

Titration of polyplexes complexed with pEGFP-N1 (1 μg) was performed in MCF-7 and SKBR-3 cells. Before polyplexes were added, the cells were treated with 10, 100, or 1,000 ng/ml at 2 × 10^4^ cells/well. After incubation for 1 h, polyplexes were added to wells in an antibiotic-free medium supplemented 10% FBS, followed by additional incubation for 36 h. GFP expression in cells was observed under a fluorescence microscope (IX71, Olympus, Japan).

### Animal experiments

To xenograft with MCF-7 cells, 5-week-old female BALB/c-nu mice (16 ± 1 g) obtained from Orient Bio Inc. (Seongnam, Gyeonggi-do, South Korea) were divided into 5 groups (*n* = 4, PBS, DPK, DPKCi, DPKCo, and DPKCu groups). Mice were housed separately with 4 animals in each cage under controlled temperature (22 ± 2 °C), humidity (55 ± 15%), and lighting (illuminance of 150 to 300 lx) conditions. MCF-7 cells (2 × 10^7^ cells/ml) were subcutaneously injected into the right flanks of the mice, which were anesthetized by inhalation of 5% (induction) and 2% (maintenance) isoflurane in pure oxygen. To compare the in vivo biodistributions of Flamma 675-labeled DPKCu/psiRNA-hBCL2 polyplexes, MDA-MB-231 and MCF-7 cells were injected into the left and right flanks in the same mouse. The samples were injected intravenously into tail veins when tumor sizes reached approximately 20 mm^3^_._ After 2 h, tumor-xenografted nude mice were imaged using a fluorescence imaging system (FOBI, Cellgentek, Cheongju, Chungcheongbuk-do, South Korea) in near-infrared (NIR) light with filtration. Hearts, kidneys, livers, lungs, and spleens were collected from sacrificed mice after 24 h, and organ distributions were determined by imaging. Fluorescence intensity of each organ harvested was calculated and converted to rainbow images using NEOimage software [7].

To exam antitumor effects of polyplexes, the copolymers were complexed with 10 μg of psiRNA-hBCL2 at 2:1 (w/w), and then the polyplexes were intravenously injected into tail veins when tumor diameters reached 4.4 to 5.2 mm^3.^ Tumor sizes were monitored for 21 d, and the mice were euthanized by CO_2_ inhalation. The excised tumor tissues were fixed in 4% paraformaldehyde, embedded in paraffin, stained by hematoxylin and eosin and BCL2 immunohistochemistry, followed by observation under a fluorescent microscope.

### Statistical analysis

Data were analyzed using Excel software and presented as the mean ± SD. Comparisons among groups were performed using one-way analysis of variance Tukey’s test of variance. **P < 0.05 was considered statistically significant.

## Results

### Identification of curcumin-binding membrane protein in MCF-7 cells

**Fig. 1.**
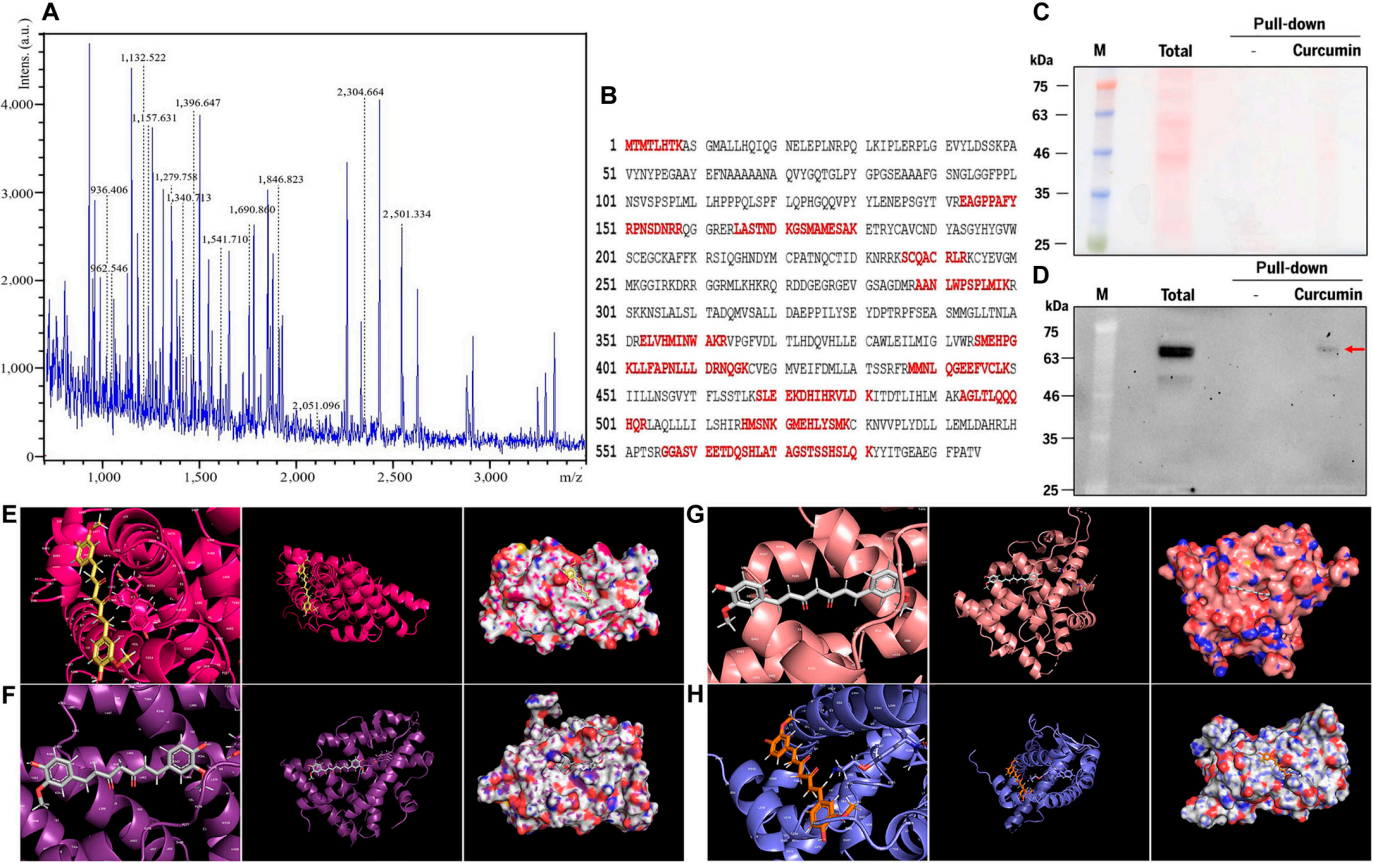
Specific binding of curcumin to ER. Pull-down assay of MCF-7 cell membrane extraction on curcumin-displayed Sepharose 4B resin. (A and B) The curcumin-binding protein was identified as ERα by matrix-assisted laser desorption/ionization time-of-flight analysis. (C and D) Curcumin-binding proteins were analyzed on SDS-PAGE (C) and western blotting (D). (E to H) Molecular docking of curcumin to ERβ (E and F) and ERα (G and H). The structures of ERβ (PDB: 4J26 [A]; 5Toa [B]) and ERα (PDB: 7qvj [C]; 8duk [D]) were docked with curcumin (PDB: CC9) in a Patch Dock server.

The cell membrane fraction was isolated from cultured MCF-7 cells, and membrane proteins were extracted. Subsequently, these proteins were applied to a Sepharose 4B resin conjugated with curcumin, and the binding proteins were eluted. The eluted proteins were separated by 2D electrophoresis, and matrix-assisted laser desorption/ionization time-of-flight analysis was performed on strongly expressed spots (Fig. 1A). Among receptors on the MCF-7 cells, an ER was found to have the strongest affinity for curcumin (Fig. 1B). To clarify this result, fractions from the pull-down assay were subjected to SDS-PAGE (Fig. 1C), and western blotting was performed using human ERα antibody (Fig. 1C). A strong band of ERα appeared in the total proteins, and it bound to the curcumin-conjugated resin but did not bind to the curcumin-free resin (red arrow in Fig. 1D). To confirm whether curcumin can bind to ERs, we attempted molecular docking of curcumin in 2 ERα (Fig. 1E and F) and ERβ (Fig. 1G and H) structures. Interestingly, unlike tamoxifen and peptides known to bind to ERs, which have existing binding sites inside the protein, curcumin was found to have binding pocket outside the protein.

### Designation and characterization of synthesized copolymers

**Fig. 2.**
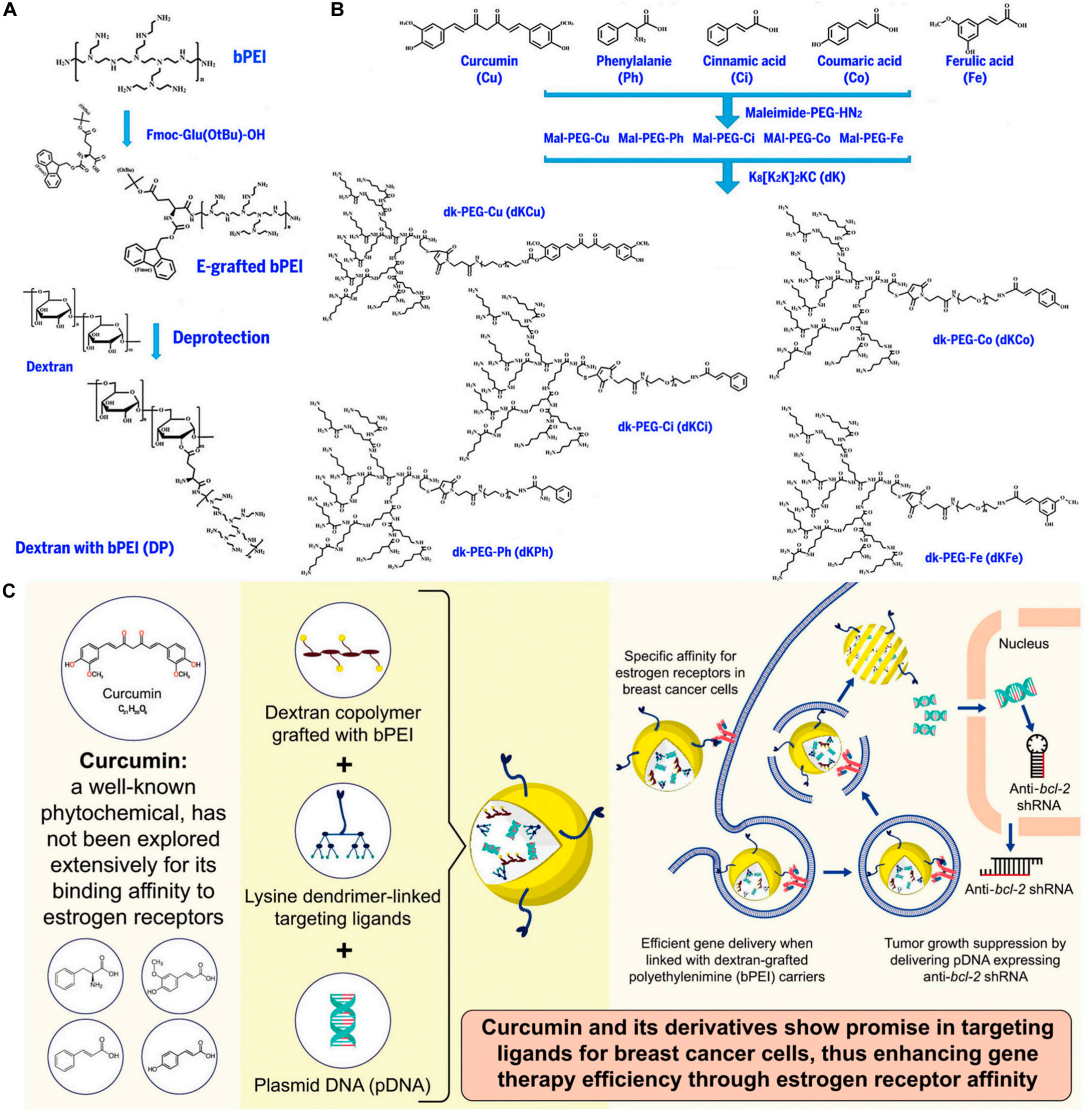
Synthesis and scheme of the targeted gene delivery system proposed in this study. (A) Synthetic procedures of dextran with bPEI (DP) copolymer by chemical reactions. After conjugation between Fmoc-Glu(OtBu)-OH amino acids and bPEI (Mw 2 kDa) (EP), OtBu protecting groups were removed by 95% TFA. Free carboxyl groups of EP were grafted to hydroxyl groups of dextran via esterification. DP copolymers were achieved by Fmoc elimination. (B) Synthetic procedures of targeting ligand-grafted lysine dendrimer peptide. (A) After PEGylation of _L_-phenylanine (Ph), trans-cinnamic acid (Ci), p-coumaric acid (Co), or trans-ferulic acid (Fe), it was grafted to lysine dendrimer peptide (K_8_[K_2_K]_2_KC, dk) via a facile maleimide-thiol “click” reaction. For curcumin-conjugation, CDI-activated curcumin was easily conjugated with Maleimide-PEG-NH_2_, followed by maleimide-thiol “click” reaction with dK peptide. (C) Nanoparticles were formed by ternary complexing of DP, targeted ligand-grafted lysine dendrimer peptide, and pDNA expressing shRNA for *bcl-2* gene. Nanoparticles were specifically targeted to ER-positive tumor cells, followed by receptor-mediated endocytosis, endosomal escape, and dissociation of nanoparticles. Plasmid DNA containing anti-*bcl-2* shRNA was localized into nucleus and expressed anti-*bcl-2* shRNA, after which siRNA can silence *bcl-2* mRNA in tumor cells.

The bPEI2K was grafted onto dextran (DP) at 1% molar ratio to bind electrostatically with pDNA because uncharged dextran cannot be complexed with pDNA (Fig. 2A). To graft bPEI2K onto dextran, we first conjugated Fmoc-Glu(OtBu)-OH to bPEI2K through a chemical reaction (E-grafted bPEI), OtBu groups were removed, and the hydroxyl groups of dextran were conjugated with the carboxyl groups of E-grafted bPEI via esterification. Ester bonds are biocompatible linkages easily cleaved by cellular esterase. The DP copolymer was obtained by Fmoc deprotection (Fig. 2A).

PEGylated curcumin and its analogs (phenylalanine, cinnamic acid, coumaric acid, and ferulic acid) was conjugated to lysine dendrimer peptides with cysteine residue (K_8_[K_2_K]_2_KC, dk) through a maleimide-thiol “click” reaction (Fig. 2B). Although PEGylated targeting ligands can be directly grafted onto DP, they were conjugated to dK peptides because these peptides can also bind with nucleic acids. Unexpected reactions due to multiconjugations can be minimized, and the percentage of targeting ligands employed is easily controlled by mixing DP and dKs. Furthermore, the present study aimed to investigate a favorable structure for specific targeting of tumor cells, including structures with phenylalanine, cinnamic acid, coumaric acid, ferulic acid, and curcumin. Successful synthesis was confirmed by ^1^H-NMR structural analysis of the products at each synthesis step (Figs. S1 to S5).

Figure 2C shows predicted mechanisms underlying effects of the targeted gene therapies developed in this study. Nanoparticles were formed by ternary complexing of DP, a targeted ligand-grafted lysine dendrimer peptide, and pDNA expressing shRNA for the bcl-2 gene, which targets the membrane ER (mER) in breast cancer cells. These particles were predicted to be internalized by receptor-mediated endocytosis, followed by endosome formation and endosomal escape by the proton sponge effect. After the nanoparticles are dissociated and pDNA is released, pDNA is localized to the nucleus, and anti-bcl-2 shRNA is expressed. Cytosolic small interfering RNA (siRNA) processing is achieved by Dicer, which silences bcl-2 mRNA.

### Nanoparticle formation of pDNA/copolymer complexes

**Fig. 3.**
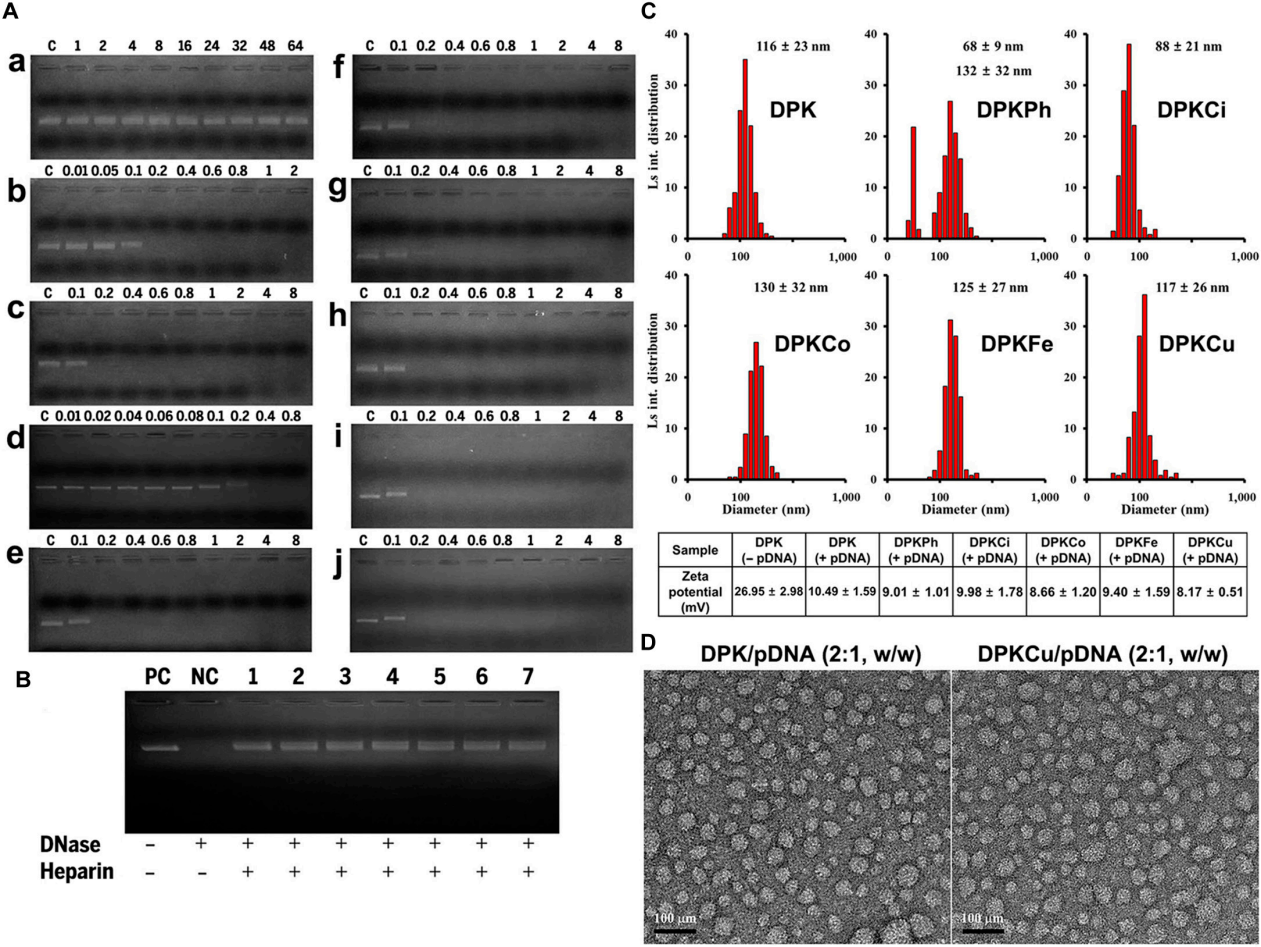
Characterization of copolymers in the presence of pDNA. (A and B) Electron mobility shift assay (A) and DNase protection assay (B) of pDNA/copolymers. (A) Dextran (a), bPEI2K (b), DP (c), dK (d), DPK (e), and DP mixed with dKPh (DPKPh) (f), dKCi (DPKCi) (g), dKCo (DPKCo) (h), dKFe (DPKFe) (i), or dKCu (DPKCu) (j) at a 9:1 ratio (w/w) was complexed with pDNA at the indicated weight ratios. (B) DP (1), DPK (2), DPKPh (3), DPKCi (4), DPKCo (5), DPKFe (6), and DPKCu (7) at a 9:1 ratio (w/w) were mixed with pDNA at a weight/weight ratio of 2:1. After incubation for 20 min at room temperature, DNase I was incubated for 20 min and then inactivated by heating at 75 °C, followed by treatment of heparin (60 units) for 10 min. (C) Size distributions and zeta potentials of DPKs/pDNA polyplexes. DPK, DPKPh, DPKCi, DPKCo, DPKFe, or DPKCu mixed at a weight ratio of 9:1 (DP:dKs) was complexed with pEGFP-N1 at a weight ratio of 2:1, followed by analysis using DLS. (D) DPK/ pEGFP-N1 or DPKCu/pEGFP-N1 was observed under transmission electron microscopy.

To investigate the binding capacity of the copolymers to pDNA, we performed an electrophoretic mobility shift assay after complexing at various weight ratios. Dextran did not bind to pDNA even at a weight ratio of 64:1 because it has only hydroxyl groups (Fig. 3A, a), whereas bPEI2K bound strongly to DNA at a weight ratio of 0.2:1, because it has many amine groups (Fig. 3A, b). The bands of pDNA/DP polyplexes displayed complete condensation at a weight ratio of 0.2:1, although only 1% bPEI2K was grafted onto dextran (Fig. 3A, c). The DP and dK peptides with targeting ligands containing phenylalanine, cinnamic acid, coumaric acid, ferulic acid, and curcumin were mixed at a weight ratio of 9:1, followed by complexation with pDNA. dK, a lysine dendrimer peptide, and DPK, a mixture of dK/DP (weight ratio of 1:9), were completely complexed at weight ratios of 0.4:1 and 0.2:1, respectively (Fig. 3A, d and e, respectively). DNA migration was completely retarded in the presence of DPKPh, DPKCo, DPKFe, and DPKCu carriers at a weight/weight ratio of 0.2:1 or 0.4:1 (Fig. 3A, f to j, respectively).

For gene delivery in vivo, an effective carrier must protect nucleic acids from degradation by nucleases in the serum and extracellular matrix. Therefore, the DNase-protecting ability of the copolymers was investigated by adding DNase I, inactivating DNase I, and treatment with heparin. A negative control treated with only DNase I was completely digested, but pDNA complexed with DP, DPK, DPKPh, DPKCi, DPKCo, DPKFe, and DPKCu at a weight/weight ratio of 2:1 showed clear DNA bands, as did the positive control, indicating protective ability against nuclease digestion (Fig. 3B).

All polyplexes were complexed with pDNA at a weight ratio of 2:1, and the mean diameter and zeta potential of DPKs/pDNA polyplexes were evaluated by dynamic light scattering (DLS) (Fig. 3C). The sizes of DPK, DPKCi, DPKCo, DPKFe, and DPKCu polyplexes with pDNA at a weight ratio of 2:1 were 116 ± 23, 88 ± 21, 130 ± 32, 125 ± 27, and 117 ± 26 nm, respectively, in unimodal form. The DPKPh/pDNA polyplex was sized in dimodal nanoparticles of 68 ± 9 and 132 ± 32 nm. The zeta potential of DPK and DPK/pDNA was 26.95 ± 2.98 and 10.49 ± 1.59 mV, respectively. The surface of DPK polyplexes with ligands had weakly positive charge (Fig. 3C). As shown in Fig. 3D, DPK/pDNA and DPKCu/pDNA polyplexes formed round nanostructures at a weight ratio of 2:1, and their observed sizes were consistent with the DLS results.

### Transfection efficiency of polyplexes with pDNA

**Fig. 4.**
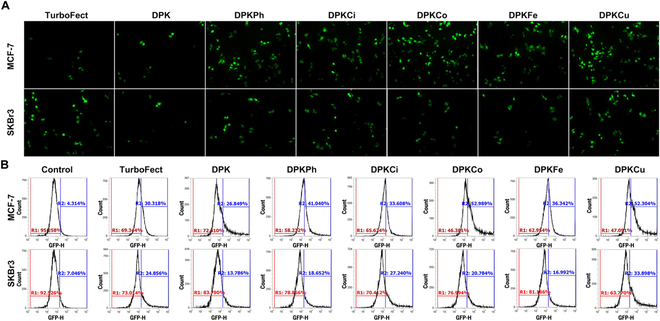
Transfection efficiency of polyplexes in MCF-7 and SKBr3 cells. (A and B) Polyplexes complexed with pEGFP-N1 at 3 formulations (5%, 10%, and 20% dK, without or with targeting ligands) and 2 weight/weight ratios of 1:1 and 2:1 were incubated for 48 h in MCF-7 and SKBr3 cells. GFP expression was visualized under an inverted fluorescence microscope (A) and evaluated by using a flow cytometry (B).

To compare the gene delivery capacity of copolymers, we complexed pDNA with DPKs, which were formulated with a dK of 5%, 10%, or 20% (w/w), at 2 weight ratios of 1:1 and 2:1, in the 2 breast cancer cell lines MCF-7 and SKBr3 (Fig. 4 and Figs. S6 and S7). The pEGFP-N1-tranfected MCF-7 cells observed by fluorescence microscopy (Fig. 4A) and cytometry analysis (Fig. 4B) showed that enhanced GFP (EGFP) expression of DPKCi, DPKCo, and DPKCu polyplexes was higher than that of DPK, DPKPh, and DPKFe at a weight/weight ratio of 2:1. All polyplexes in the presence of targeting ligands exhibited potent transfection efficacy at a weight/weight ratio of 2:1 when compared to that of DPK. In addition, fluorescence intensities of the microtiter wells were measured to normalize gene transfection efficiency (Fig. S6). Polyplexes with 10% (w/w) targeting ligands showed the strongest EGFP expression, and those complexed with DPKCu emitted the highest green fluorescence. Although EGFP expression in SKBr3 cells was higher than that in MCF-7 cells, transfection efficiency in SKBr3 cells was similar to that in MCF-7 cells, except for the potent green fluorescence intensity of DPK polyplexes in all tested formulations in SKBr3 cells (Fig. S7).

In an investigation of gene transfection ability in various cancer cells, MDA-MB-231 (breast cancer), SKOV-3 (ovarian cancer), PC3 (prostate cancer), and DLD-1 (colon cancer) cells transfected with polyplexes in the presence of 10% (w/w) targeting ligands showed no marked targeting capacity in any of the cell lines (Fig. S8). Figure S9 shows the gene transfection efficiency of the polyplexes in HaCaT cells, which are a normal cell line.

### Cellular uptake and cellular distribution

**Fig. 5.**
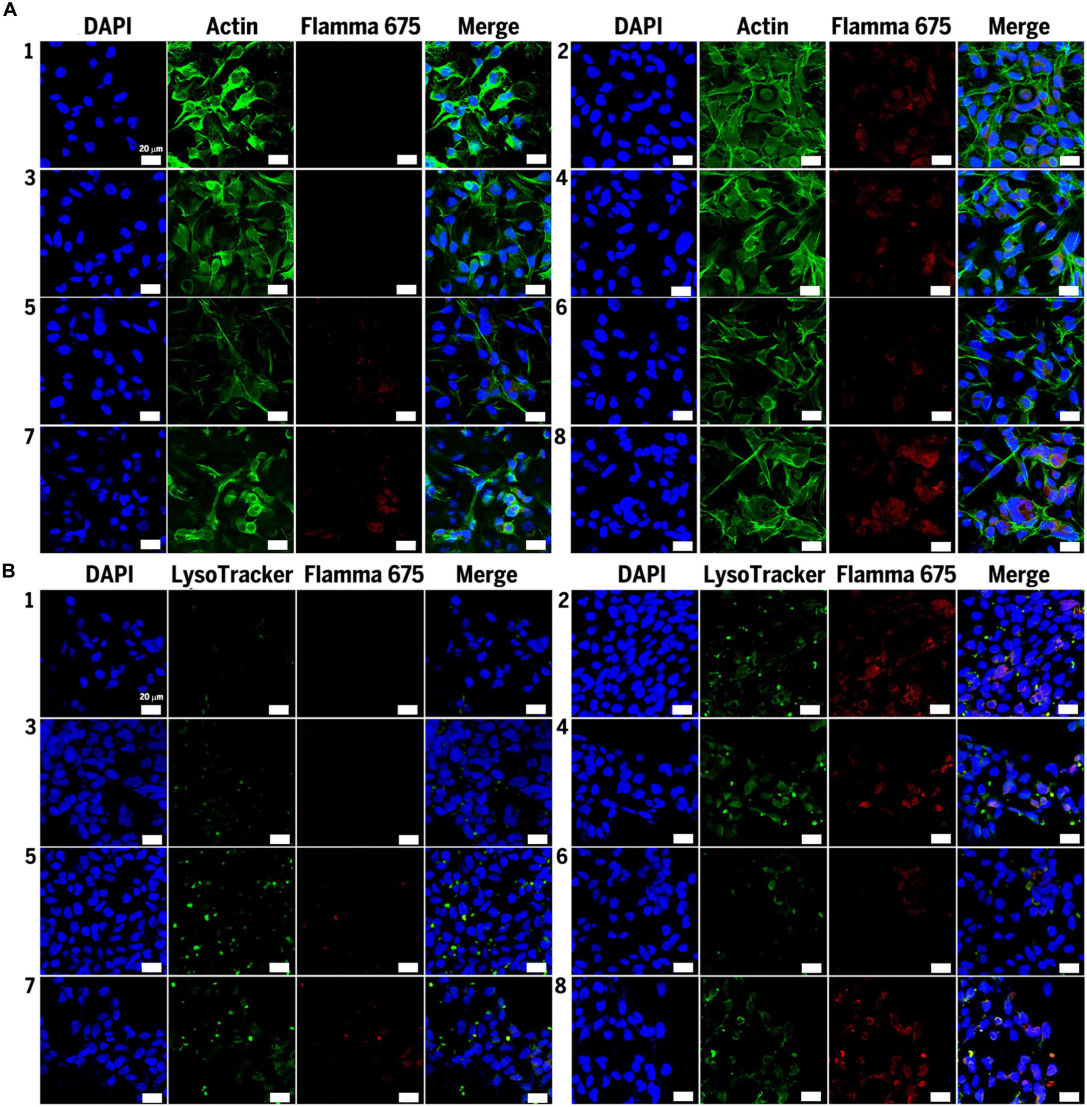
Cellular uptake and endosomal escape of Flamma 675-labeled polyplexes in MCF-7 cells. (A and B) Flamma 675-labeled DP mixed with unlabeled DP at 1:9 ratio (w/w) were complexed with dK derivatives and pET28(a) (copolymers at 9:1 w/w), pDNA at 2:1 w/w), followed by a 4-h (A) or 6-h (B) incubation. Fixed cells were stained with ActinGreen 488 (green [A]), LysoTracker (green [B]), and DAPI (blue) and visualized under confocal laser scanning microscopy. 1: control, 2: pET28(a)/PEI2K, 3: pET28(a)/DPK, 4: pET28(a)/DPKPh, 5: pET28(a)/DPKCi, 6: pET28(a)/DPKCo, 7: pET28(a)/DPKFe, and 8: pET28(a)/DPKCu.

To investigate the cellular uptake and distribution of pDNA/copolymer complexes, we conjugated Flamma 675 fluorescence dye to the amine groups of DP, and unlabeled DP and labeled DP were mixed at a weight/weight ratio of 9:1 to minimize artificial effects of the conjugated fluorescent dye. The mixture was complexed with dKs containing targeting ligands (9:1, w/w) and pET28 DNA (a non-GFP expression vector), and the polyplexes were transfected into MCF-7 cells. Sections of the cytosol and nucleus were stained with specific actin (ActinGreen 488 probe, green fluorescence) and nuclear (DAPI probe, blue fluorescence) antibodies. Figure 5A shows that strong red fluorescence was notable after transfection with DPKCi, DPKCo, and DPKCu polyplexes but that PEI2K polyplexes were not internalized. Intracellular fluorescence after DPK, DPKCo, and DPKFe transfection was low. These results suggest that the targeting ligands DPKCi, DPKCo, and DPKCu have a specific binding affinity for ERs in the plasma membrane of MCF-7 cells.

Furthermore, LysoTracker (green fluorescence) and DAPI were used to detect endosomes and nuclei, respectively. As shown in Fig. 5B, the red fluorescence of DPK, DPKPh, and DPKFe polyplexes exactly overlapped with the green fluorescence, indicating limited localization of polyplexes within endosomes, although low intracellular localization was observed in MCF-7 cells compared to that of DPKCi, DPKCo, and DPKCu. In contrast, red fluorescence of the DPKCi, DPKCo, and DPKCu polyplexes showed that cellular was inconsistent, suggesting early endosomal escape in some cases.

### Dose-dependent inhibition of gene transfection after preincubation of curcumin or estradiol

**Fig. 6.**
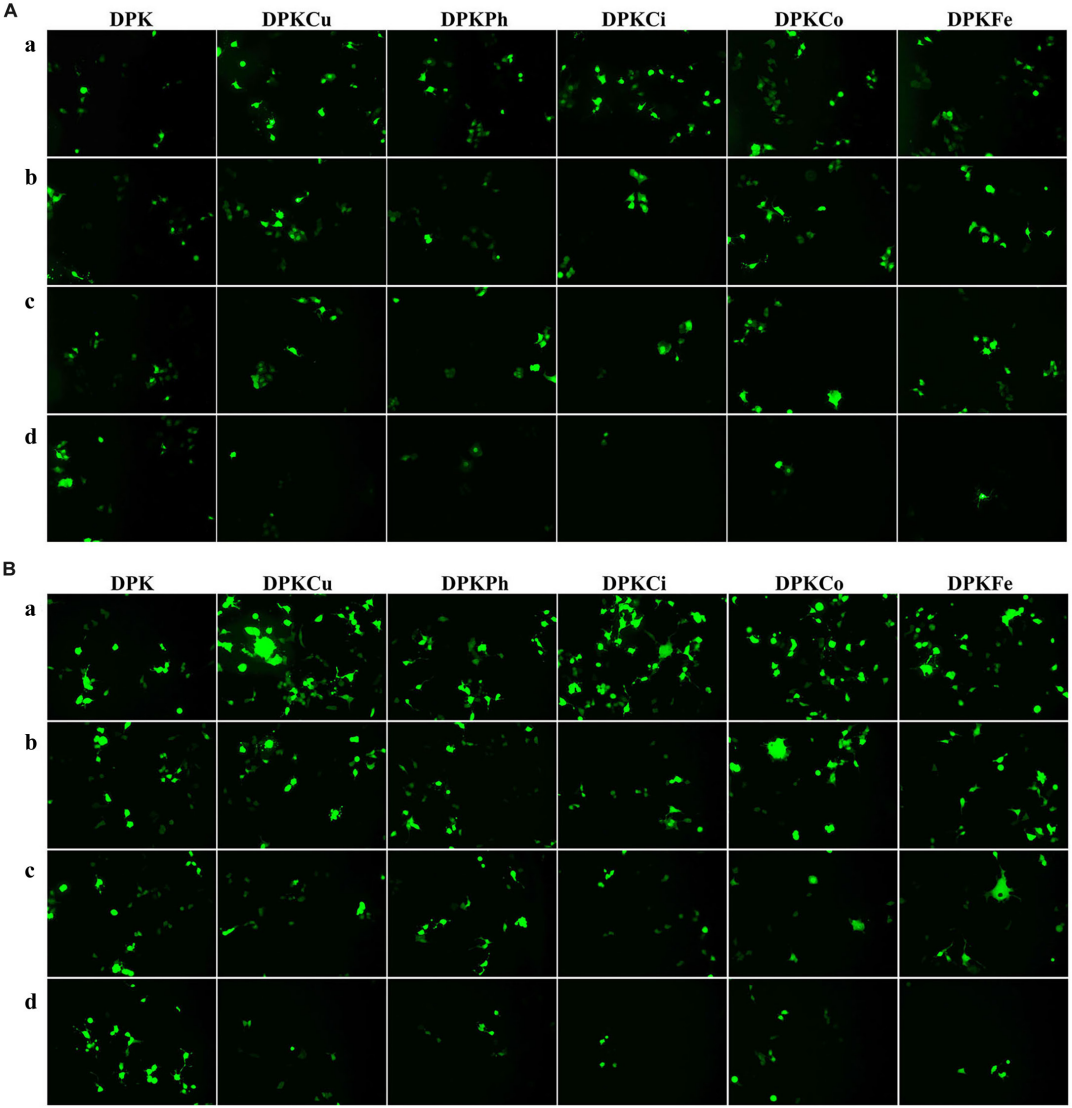
Dose-dependent inhibition of gene transfection via preincubation of curcumin in breast cancer. (A and B) After nontreatment (a) or treatment with 10 ng/ml (b), 100 ng/ml (c), or 1,000 ng/ml curcumin (d) for 1 h in MCF-7 (A) or SKBr3 (B), polyplexes (1:2, w/w) were incubated with pEGFP-N1 for 36 h.

The gene transfection efficacy of polyplexes was analyzed after preincubation with curcumin or 17α-estradiol, a ligand for ERs. As shown in Fig. 6, preincubation with curcumin inhibited gene transfection via DPKPh, DPKCi, DPKCo, DPKFe, and DPKCu in a dose-dependent manner in both MCF-7 (Fig. 6A) and SKBr3 (Fig. 6B) cells. However, the transfection efficiency of DPK was not altered by curcumin in either cancer cell line. Figures S10 and S11 show that the transfection of DPKPh, DPKCi, DPKCo, DPKFe, and DPKCu polyplexes were inhibited in the presence of 17α-estradiol, an inhibitor of ER, in MCF-7 cells. These results suggested that MCF-7 and SKBr3 cells have one targetable receptor, at least in the plasma membrane. Therefore, this study focused on mERs as the target for curcumin.

### Cytotoxic and antitumor effects of polyplexes

**Fig. 7.**
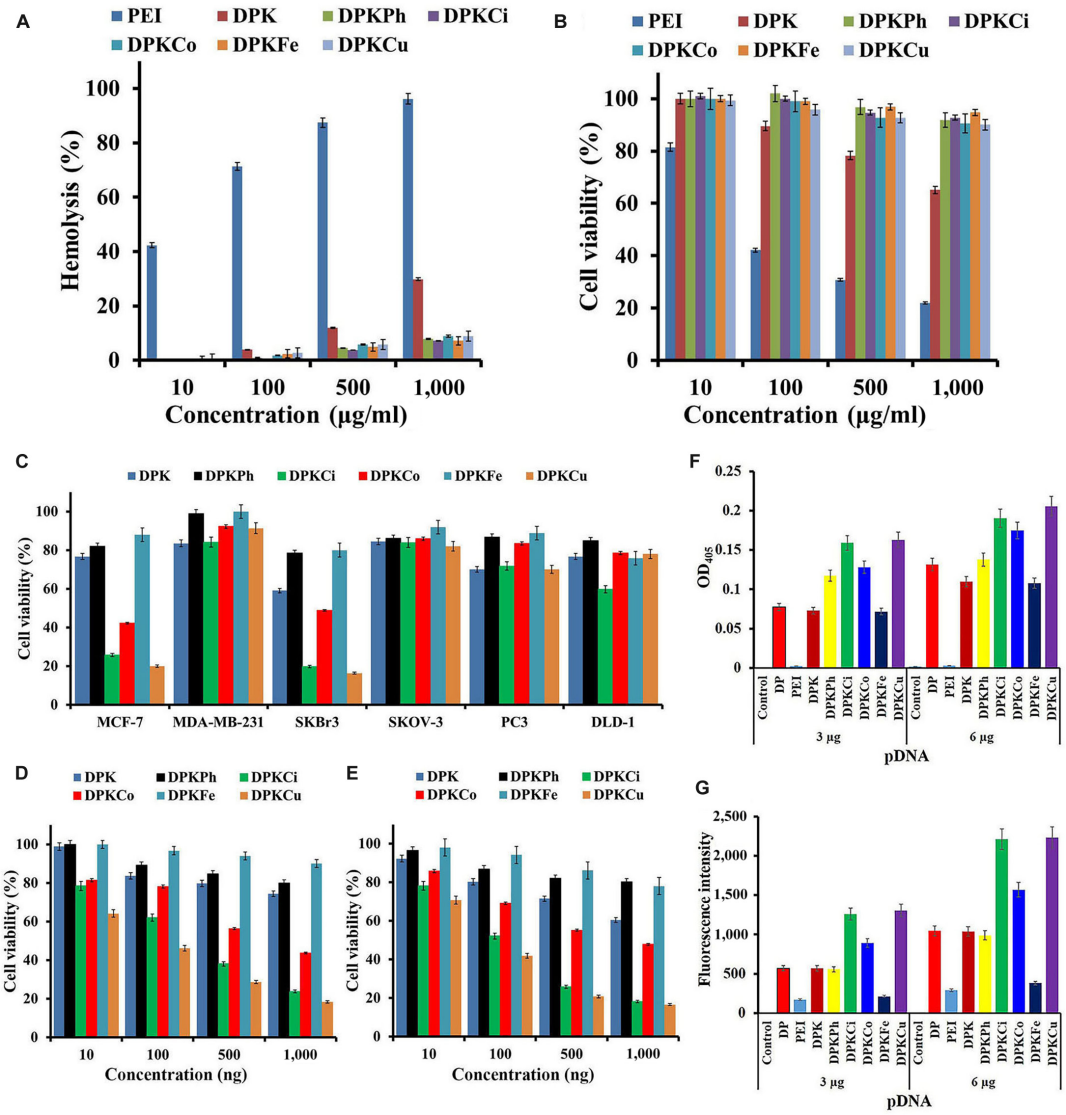
Hemolytic, cytotoxic, and antiumor effects of polyplexes in rat erythrocytes, HaCaT cells, and various tumor cells. (A and B) After 1- and 48-h incubations of polyplexes complexed at indicated concentrations of psiRNA-hBCL-2 with rRBC (A) and HaCaT (B) cells, respectively, released hemoglobin and cell viability were measured. Antitumor effects of polyplexes with psiRNA-hBCL2 in various cancer cells. (C) After complexing of 1 μg of psiRNA-hBCL2 with copolymers (w/w, 2:1), polyplexes were incubated with MCF-7, MDA-MB-231, SKBr3, SKOV3, PC3, and DLD-1 cells for 48 h. (D and E) Polyplexes with psiRNA-hBCL2 (10, 100, 500, or 1,000 ng) (w/w, 2:1) were subjected to MCF-7 (D) or SKBr3 (E), and the cells were incubated for 48 h, followed by CCK-8 assay. (F and G) Upregulated caspase-9 (F) and caspase-3 (G) levels in the presence of polyplexes with psiRNA-hBCL2 in MCF-7 cells. After incubation of polyplexes with pDNA (3 or 6 μg) at weight/weight ratio of 2:1, intracellular caspase-9 and caspase-3 were quantitatively assayed using an enzyme-linked immunosorbent assay method.

To evaluate safety of the polyplexes in normal cells, we performed hemolysis and cytotoxicity assays using rRBCs and HaCaT cells (human keratinocytes). As shown in Fig. 7A, the hemolysis rates of all copolymers except for PEI and DPK were less than 9% at 1,000 μg/ml, but that of DPK was 29.87% at 1,000 μg/ml. The pDNA/PEI2K complex induced a remarkable hemoglobin release from rRBC, even at the 10 μg/ml concentration, which was the experimental minimum (Fig. 7A). We assessed cytotoxicity of the polyplexes in HaCaT cells using the CCK-8 assay. PEI polyplexes with 100 μg/ml pDNA showed 42.1% cell viability, indicating highly cytotoxic effects (Fig. 7B). The percentage cell survival with pDNA/DPK polyplexes was 65.11% at 1,000 μg/ml, whereas that with other copolymers and pDNA was more than 90% at 1,000 μg/ml.

A psiRNA-hBCL2 pDNA expressing shRNA for silencing the *bcl-2* gene was used to demonstrate antitumor effects after targeted gene delivery. These effects were evaluated by CCK-8 assay in MCF-7, MDA-MB-231, SKBr3, SKOV-3, PC3, and DLD-1 cancer cells. As shown in Fig. 7C, cell viabilities following delivery of psiRNA-hBCL2 was remarkably reduced in MCF-7 and SKBr3 cells. This result suggests that curcumin and its derivatives possess specific binding or targeting abilities for mER. To compare effects of targeting-ligands on antitumor effects, we performed dose-dependent gene transfections in MCF-7 (Fig. 7D) and SKBr3 (Fig. 7E) cells. The results showed that cell viability was dose-dependently reduced in the presence of DKPCi and DPKCu, which is consistent with the results of transfection efficiencies.

To ascertain the induced apoptosis resulting from psiRNA-hBCL2 delivery, we measured overexpression of caspase-9 and caspase-3 (Fig. 7F and G), which are molecules in the apoptotic cascade, by enzyme-linked immunosorbent assay in MCF-7 cells. Caspase-9 was upregulated by transfection with the pDNA/DPKCi, pDNA/DPKCo, and pDNA/DPKCu polyplexes, and caspase-3 was remarkably overexpressed following delivery of pDNA/DPKCi and pDNA/DPKCu. These results were consistent with those of transfection efficiency.

### In vivo targeted gene delivery and antitumor effects of DPKCu polyplexes

**Fig. 8.**
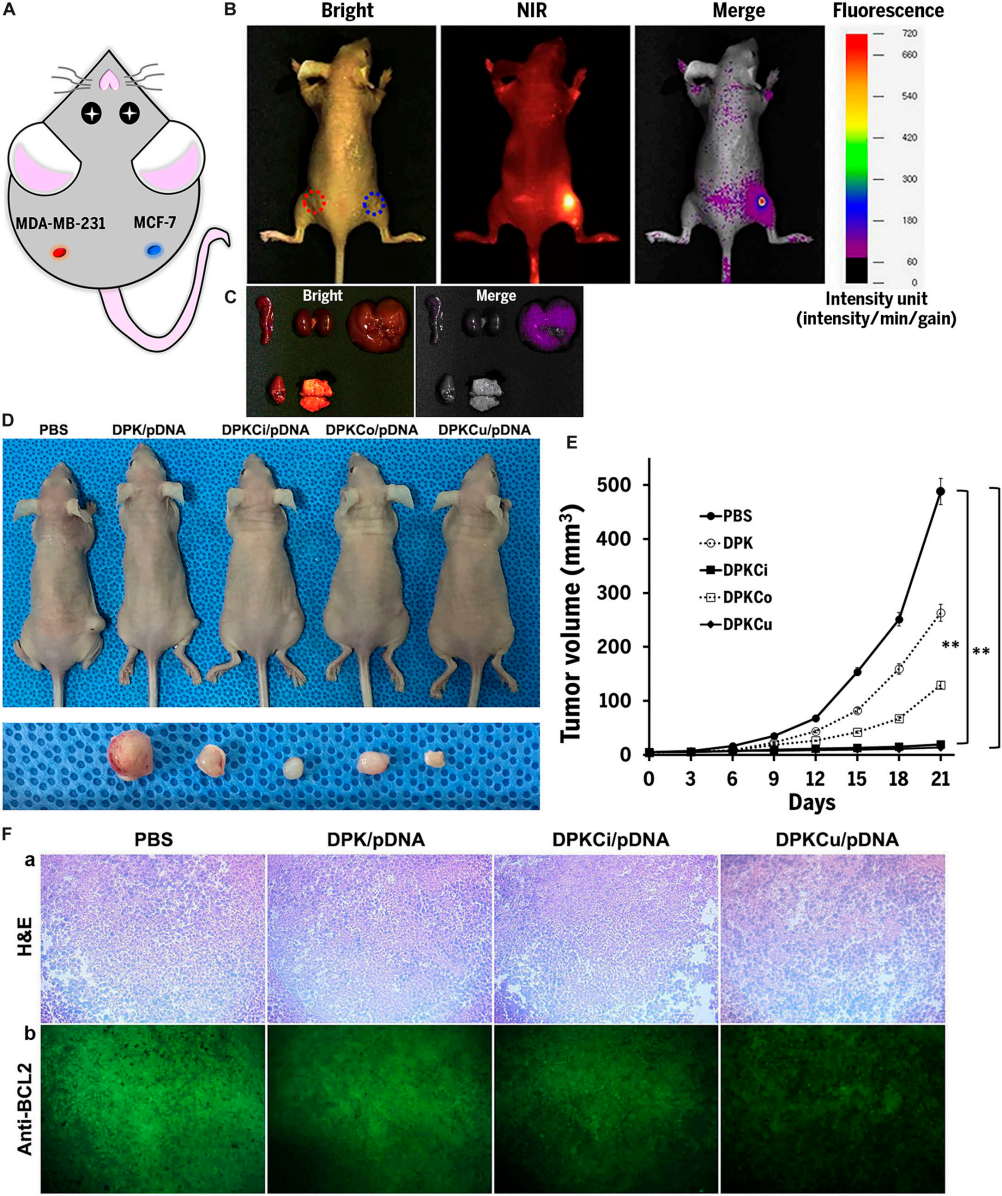
In vivo antitumor effects of polyplexes via targeted gene delivery. (A and B) In vivo biodistribution of Flamma 675-labeled DPKCu/pDNA (w/w 2:1) polyplexes. In vivo bright, NIR, and merged images of mouse double-xenografted with MDA-MB-231 and MCF-7 cancer cells were observed at 2 h after intravenous injection with polyplexes. (C) After 24 h, mice were sacrificed and organs (liver, lung, kidney, heart, and spleen) extracted. Bright and NIR images of organs were merged. (D) Images of whole tumor-bearing mice and excised tumors on day 21 after treatments. PBS, DPK/psiRNA-hBCL2 (w/w, 2:1), DPKCi/psiRNA-hBCL2 (w/w, 2:1), DPKCo/psiRNA-hBCL2 (w/w, 2:1), and DPKCu/psiRNA-hBCL2 (w/w, 2:1). (E) Distribution of time-dependent tumor volumes. Tumor volumes were calculated as tumor volume = (width)^2^ × length × 1/2. **P < 0.05 versus PBS-treated control. (F) Hematoxylin and eosin (H&E) (a) and immunohistochemical analysis (b) of BCL2 protein in harvested tumors (×200).

To prove the tumor-homing capacity of curcumin, we intravenously injected Flamma 675-labeled DPKCu/psiRNA-BCL2 (w/w, 2:1) polyplexes into MDA-MB-231 (left flank) and MCF-7 (right flank) double-xenografted mice (Fig. 8A) and observed them using a NIR optical imaging system. After 2 h, a remarkable NIR signal in tumor-bearing mice was observed on the MCF-7-xenografted flank but was not detected on the MDA-MB-231-xenograted flank (Fig. 8B). This NIR signal weakened slightly over time but was maintained for 96 h (data not shown). Polyplexes were injected into the mouse tail vein; 24 h later, major organs (heart, kidney, liver, lung, and spleen) were removed, and biodistributions of the polyplexes were confirmed by observing NIR fluorescence. The NIR signal appeared in the liver but not in other organs (Fig. 8C).

To confirm antitumor effects in vivo, polyplexes were formed with 10 g of psiRNA-hBCL2 using DPK, DPKci, DPKCo, or DPKCu (w/w, 9:1) at a weight ratio of 2:1 and injected into MCF-7-xenografted mice. As shown in Fig. 8D, statistically significant tumor growth inhibition was observed in the groups treated with DPKCi and DPKCu polyplexes. In comparison to the PBS group, although cancer masses were inhibited by administration of DPK and DPKCo polyplexes, cancer formation was progressive. At 21 d after sample injection, tumor masses of the PBS-administered mice increased to 488 mm^2^, whereas those from mice administered DPK polyplexes showed a mean mass size of 263 mm^2^. Mice treated with DPKCi, DPKCo, or DPKCu polyplexes had average tumor masses of 20, 130, or 14 mm^2^, respectively (Fig. 8E). Figure 8F shows histological analysis with hematoxylin and eosin and immunohisochemical analysis with anti-Bcl-2 protein. The administration of DPKCi and DPKCu polyplexes remarkably reduced the cancer cells in the tumor tissue and inhibited Bcl-2 protein expression, compared to the PBS-treated group.

## Discussion

Breast cancer is the second most common cancer in the worldwide, with approximately 2.26 million incidences in 2020, and one of the main cancer-related mortality in women [29]. Several risk factors, such as family history, genetic mutations, aging, estrogen, sex, and lifestyle, increase the incidence of breast cancer. It is a metastatic cancer that can spread to organs such as the liver, lungs, bones, and brain after its onset [29]. It is mainly classified into 3 main types by the presence of human epidermal growth factor 2, estrogen or progesterone receptor, and triple-negative breast cancer, lacking all 3 standard molecular markers [30]. Early diagnosis by glycoproteins, blood angiogenic factors, hormone receptors, and other potential biomarkers increases survival rates. Approximately 50% to 60% of primary breast cancer lesions are diagnosed with ER overexpression (ER-positive). Two classes of ER exist: intracellular ERs (ERα and ERβ) regulate transcription driving growth, proliferation, and differentiation by estrogen signaling; mERs (GPR30, ER-X, and G_q_-mER) are mostly G protein-coupled receptors [31,32]. Present study focused to verify the potential of curcumin as a specific targeting ligand in drug delivery via screening ER as a curcumin-binding protein among membrane proteins of MCF-7.

A variety of studies are being conducted to deliver antitumor drugs such as genes, chemical drugs, and peptides to tumor tissues and cells using nanoparticles. Although the size of the endothelial cell gap and the cross-endothelial channels affect the exudation strength of nanoparticles [33–35], early studies have explored systems for delivering drugs to tumor tissue by the enhanced permeability and retention (EPR) effect. However, this passive targeting does not have a marked therapeutic effect because it is difficult to obtain a sufficiently high drug concentration to manage tumor tissue [36,37]. The active targeting strategy of nanoparticles is mainly focused on the overexpression of many receptors such as epidermal growth factor, folate, transferrin, and integrin receptor present on the surface of vascular endothelial cells of cancer cells or tumors compared to normal cells [38,39]. In general, active tumor targets are membrane proteins, which are overexpressed on tumor, angiogenic endothelial, malignant, or inflammatory cells. There are approximately 7,000 known transmembrane proteins in cells, and ~150 of them are overexpressed in tumor cells or tumor-associated cells. They are potential drug or imaging diagnostic targets. Overexpression of special receptors can increase specific recognition between the nanoparticle and cancer cells by displaying various types of ligands, such as antibodies, aptamer, peptides, and polysaccharides, on the nanoparticle surface, being increasingly used for active targeting. Therefore, finding various ligands that can specifically recognize tumor tissues or cells is very important for effective cancer treatment through active targeting.

Curcumin, a natural polyphenolic phytoalexin isolated from *Curcuma longa*, has a broad spectrum of biological effects [40]. Recently, its anticancer effect was discovered after ingestion as a food or injection of its nanoformulation into the body [24]. Elucidating the molecular mechanism by interfering multiple cell signaling pathways such as regulating cell cycle (cyclin D1 and cyclin E), apoptosis (caspase and down-signaling gene products), proliferation (human epidermal growth factor 2, epidermal growth factor receptor, and activating protein-1), survival (phosphoinositide 3-kinase/protein kinase B), invasion (matrix metallopeptidase-9), angiogenesis (vascular endothelial growth factor), metastasis (CXC-chemokine receptor-4), and inflammation (tumor necrosis factor, interleukin- 1, interleukin-8, interleukin-12, nuclear factor-kappa B, cyclooxygenase-2, and 5-lipoxygenase) holds potential of the development of novel cancer treatments [40]. In addition, cinnamic acid inhibited human ovarian, colon, and breast cancer cell proliferation [41,42]. Ferulic acid can inhibit cell proliferation and invasion in HeLa and Caski cells by induction of apoptosis and cell cycle arrest and have antiangiogenic action [43]. p*-*Coumaric acid can inhibit the proliferation of a variety of tumor cells by causing cell cycle arrest and inducing apoptosis [44,45].

Although curcumin is a promising phytochemical with therapeutic benefits, studies elucidating its mechanism of action have been limited to intracellular targets such as reactive oxygen species, apoptosis signaling cascades, and DNA damage. We believe that curcumin must first enter cells to exert its diverse antitumor effects. If it randomly penetrates a cell membrane, it will not only exhibit greater anticancer activity but also result in serious cytotoxicity. Our research explores curcumin’s potential as a tumor-targeting ligand for gene delivery in breast cancer. We firstly identified ERs on membrane of MCF-7 cells with a high affinity for curcumin via pull-down assay. This study sought to determine whether curcumin can target cancer cells via ERs and to evaluate the antitumor efficiency of a gene delivery system with curcumin in vitro and in vivo. Therefore, to eliminate the artificial effects of antitumor drugs or drug delivery carriers, we designed a gene delivery system using dextran as a vehicle to form nanoparticles. Although charged biopolymers such as chitosan, hyaluronic acid, and alginate can be used, uncharged dextran, which is a biodegradable, biocompatible, and nonimmunogenic polysaccharide, was selected to minimize nonspecific cellular delivery via electrostatic interactions. The bPEI2K was grafted onto dextran to form a polyplex by combining with genes via electrostatic attraction, which can improve endosomal escape via the proton sponge effect when the gene carrier is endocytosed. Target ligands can be directly conjugated to dextran, but it is difficult to conjugate each of the 5 ligands in the same ratio. Therefore, we conjugated PEGylated ligands to a cationic lysine dendrimer that can electrostatically bind to genes and completed polyplexes in which they were added in equal proportions. Additionally, lysine dendrimers can also induce the proton sponge effect.

As shown in Fig. 3, when bPEI2K was bound to the surface of pDNA, a large structure and other amine groups that do not participate in formation of this complex can prevent binding of another bPEI2K complex via cationic repulsion. Because most DP amine groups were bound to pDNA and dextran was exposed on the surface of the polyplex, no other interactions occurred. Therefore, the DP copolymer was considered a suitable cationic polymer for gene carriage in this study. In addition, curcumin and 4 analogs were conjugated to lysine dendrimer peptides and used as tumor-targeting ligands of nanoparticles. The electrophoretic mobility shift assay results suggest that the targeting ligands do not interfere with pDNA binding. In addition, the protection capacity for DNase I showed that mixtures of copolymers and dK derivatives strongly bind to DNA by electrostatic interactions and maintain the stable polyplexes in blood and other body fluids. Polyplex size is an important factor in the intercellular delivery of polyplexes and in vivo intravenous injections. The size of polyplexes ranged 80 to 140 nm suggested that all nanoparticles were of a suitable size for gene delivery. Surface charge is one of the key factors in endocytosis process of gene transfection as well as the cytotoxicity. Because the membrane of cancer cells is negatively charged, cationic surface-charged nanoparticles may have a very high potential for cellular uptake.

The results of transfection efficiency in vitro are important for determining whether the targeted gene carriers developed in this study can be used in vivo. For example, if the DPKCi, DPKCo, and DPKCu complexes, which show high transfection efficiency in 2 breast cancer cell lines (MCF-7 and SKBr3), are efficiently translocated into the cytosol of normal cells, they cannot be used as targeted gene carriers in tumor cells. However, their gene transfection capacity is remarkably decreased in normal cells. These results suggest that cinnamic acid, coumaric acid, and curcumin specifically bind to or target the ERs of MCF-7 and SkBr3 cells. Furthermore, these good transfection capacity via fast binding with tumor cells through targeting ligands and early endosomal escape via polycationicity.

A transfection inhibition assay using curcumin and 17α-estradiol showed that curcumin displayed on nanoparticles specifically recognized ERs. Especially, 17α-estradiol is an endogenous stereoisomer of the hormone 17β-estradiol (E2), which targets ERs, but their affinities are different [46]. 17α-estradiol is a short-acting estrogen that forms a complex with the estrogen ligand receptor in human breast cancer cells for a short period of time. As shown in Figs. S10 and S11, almost all gene delivery into MCF-7 cells was inhibited by preincubation with 17α-estradiol, with the exception of DPK polyplex delivery. SKBr3, a recognized ER-negative breast cancer cell line, can express both ERβ1 and 2 variants, which are also expressed in most of ER-positive cancer cells [47]. A previous study proposed that G protein-coupled receptor 30 (GPR30) is related to estrogen-induced transactivation of epidermal growth factor receptor and adenylyl cyclase activity in SKBr3, which lacks nuclear ERs, and is a novel mER [31,32,46,47]. Although the relationship between curcumin and G protein-coupled ERs is not clearly understood, direct evidence to define their roles is expected from future research.

As shown in Fig. 7A and B, all polyplexes did not show remarkable toxicity or hemolysis in HaCaT cells and erythrocytes, resepctively. These results suggest that polyplexes without targeting ligands may be easily transferred into normal cells, and targeting ligands displayed on the surfaces of nanoparticles may prevent internalization across the cell membrane, which is consistent with results of the transfection in HaCaT cells. Cytotoxic and hemolytic evaluations suggested that DPK mixtures with targeting ligands are biocompatible and blood compatible. We used pDNA expressing *bcl-2* shRNA as an antitumor gene. When pDNA was delivered into the cells, an shRNA, an artificial RNA fragment with a tight hairpin turn, was continuously expressed in the nucleus of transfected cells. This shRNA had the advantages of relatively low degradation and turnover compared with siRNA. Because the *bcl-2* gene is an antiapoptotic gene that can suppress apoptosis of abnormal cells, silencing of *bcl-2* mRNA results in caspase-dependent apoptosis and autophagic cell death. The bcl-2 oncogene is overexpressed in 50% to 70% of all human cancers, as well as breast cancers [48]. Therefore, inhibition of *bcl-2* mRNA can induce sensitivity to antitumor therapies, and delivery of this shRNA into cancer cells is a promising gene therapeutic approach. Caspase activity has been implicated in the induction of mitochondrial damage during apoptosis. Activation of caspase-9 in the apoptosome through dimerization leads to loss of mitochondrial membrane potential, as well as cleavage of Bcl-2, Bcl-xL, and Mcl-1. This activation can be regulated by downstream effector caspases, including caspase-3, caspase-6, and caspase-7; pro-caspase-3 can be activated by an increase in caspase-9, followed by apoptosis [49]. In Fig. 7F and G, gene transfection with DPKCi and DPKCu showed greater activation of caspase-3 than caspase-9, suggesting that cinnamic acid and curcumin, which are released from the dissociated polyplexes after the endosomal escape of internalized pDNA/DPKCi and pDNA/DPKCu polyplexes, may activate caspase-3. This indicates synergistic induction of apoptosis following delivery of the bcl-2 shRNA and target materials (cinnamic acid and curcumin).

In vivo targeting and antitumor effects are very important results in determining whether curcumin can actually be used clinically. It is a very interesting result that the NIR signal of nanoparticles injected to double tumor-xenografted mice was strong only in the MCF-7 region. These results indicate that curcumin displayed on the surface of the polyplexes allows specific homing to tumor tissues or cells in vivo. The absence of NIR signal in other organs except the liver suggests that most of the polyplexes accumulated in the tumor mass though some polyplexes remained in the liver as a “first-pass effect”. Although more precise biotoxicity analyses need to be conducted in the future, we predict that in vivo toxicity will be minimal based on these results. Administration of polyplexes with pDNA significantly inhibited the increase in tumor masses, and a decrease in Bcl-2 protein was confirmed in immune-stained tumor tissues. These findings suggest that curcumin and cinnamic acid hold potential as homing or targeting ligands and that silencing Bcl-2 mRNA is very effective when applying gene therapies for cancer management.

In summary, this study examined whether curcumin can specifically target or home to cancerous tissues and cells in vivo, rather than having antitumor effects. In addition, the capacity of curcumin and its derivatives were compared as targeting ligands for tumor cells and tissues. Curcumin and cinnamic acid specifically target mERs in MCF-7 and SKBr3 breast cancer cells. Further, these results suggest that DPKCi and DPKCu have great potential as gene delivery systems for targeted therapy of breast cancer cells.

## Data Availability

All data required of the paper are included in the paper and/or the Supplementary Materials. The other related data may be requested from the authors.
