# Supplementary material for: Advancing Breast Cancer Therapeutics: Targeted Gene Delivery Systems Unveiling the Potential of Estrogen Receptor-Targeting Ligands
Source: Biomater Res. 2024 Sep 24;28:0087. doi: 10.34133/bmr.0087 (PMC11420687; doi:10.34133/bmr.0087)
Supplement: Supplementary 1 — Methods Figs. S1 to S11 [file bmr.0087.f1.docx]

**Supplementary Information**

Advancing breast cancer therapeutics: Targeted gene delivery systems unveiling the potential of estrogen receptor-targeting ligands

Jung Ro Lee^1,†^, Young-Min Kim^2,†^, Eun-Ji Kim^2^, Mi-Kyeong Jang^2,*^, Seong-Cheol Park^2,*^

**Methods**

**Two-dimensional differential gel electrophoresis.** IPG dry strips (4-10 NL IPG, 24cm, Genomine Co., Pohang, Korea) were equilibrated for 12-16 h with 7 M urea, 2 M thiourea containing 2% 3-[(3-cholamidopropy) dimethyammonio]-1- propanesulfonate (CHAPS), 1% dithiothreitol (DTT), 1% pharmalyte and respectively loaded with 4.8 μg (Control) and 15.0 μg (protein) of sample. Following manufacturer's instruction, isoelectric focusing (IEF) was performed at 20°C using a Multiphor II electrophoresis unit and EPS 3500 XL power supply (Amersham Biosciences, Chicago, IL, USA). For IEF, the voltage was linearly increased from 150 to 3,500 V during 3 h for sample entry, followed by constant 3,500 V, focusing entirely after 96 kVh. Prior to the second dimension, strips were incubated for 10 min in equilibration buffer (50 mM Tris-HCl, pH 6.8 containing 6 M urea, 2% SDS, and 30% glycerol), first with 1% DTT and second with 2.5% iodoacetamide. Equilibrated strips were inserted onto 10-16 % SDS-PAGE gels. Following the manufacturer's instruction, SDS-PAGE was performed using Hoefer DALT 2D system (Amersham Biosciences) SDS-PAGE. 2D gels were run at 20°C for 1,700Vh. And then, 2D gels were Colloidal CBB staining. Quantitative analysis of digitized images was carried out using the PDQuest (Bio-Rad, Hercules, CA, USA) software version 7.0 according to the protocols provided by the manufacturer. The quantity of each spot was normalized by the total valid spot intensity. Protein spots were selected for the significant expression variation that deviated over two-fold in its expression level compared with the control or normal sample. Protein spots in 2DE gel were analyzed by Genomine Co.

**Matrix Assisted Laser Desorption/Ionization Time-of-Flight Mass Spectrometry (MALDI-TOF MS).** For protein identification by peptide mass fingerprinting, protein spots were excised, digested with trypsin (Promega), mixed with α-cyano-4-hydroxycinnamic acid in 50% acetonitrile /0.1% TFA, and subjected to Autoflex Speed LRF MALDI-TOF analysis (Bruker Daltonics, Billerica, MA, USA). Spectra were collected from 300 shots per spectrum over the m/z range 700-4000 and calibrated by two-point internal calibration using Trypsin auto-digestion peaks. The peak list was generated using Flex Analysis 3.4. The threshold for peak-picking was as follows: 500 for a minimum resolution of monoisotopic mass, 5 for S/N. The search program MASCOT, developed by Matrixscience (http://www.matrixscience.com/), was used for protein identification by peptide mass fingerprinting. MALTI-TOF MS was analyzed at Genomine Co.


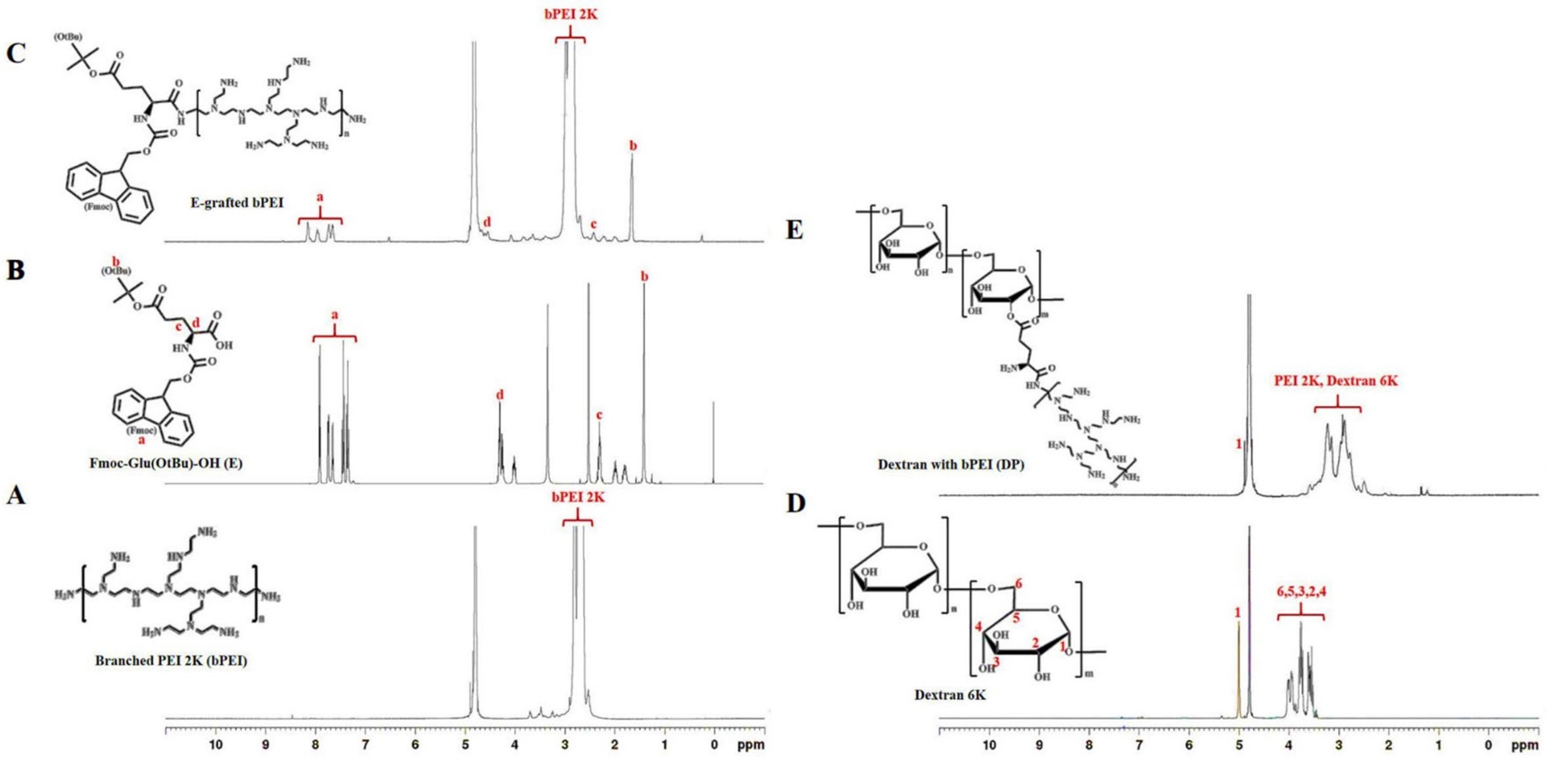


**Fig.S1.** ^1^H-NMR spectra of bPEI (A), Fmoc-Glu(OtBu)-OH (B), E-grafted bPEI (C), dextran (D), and dextran with bPEI (DP) (E).


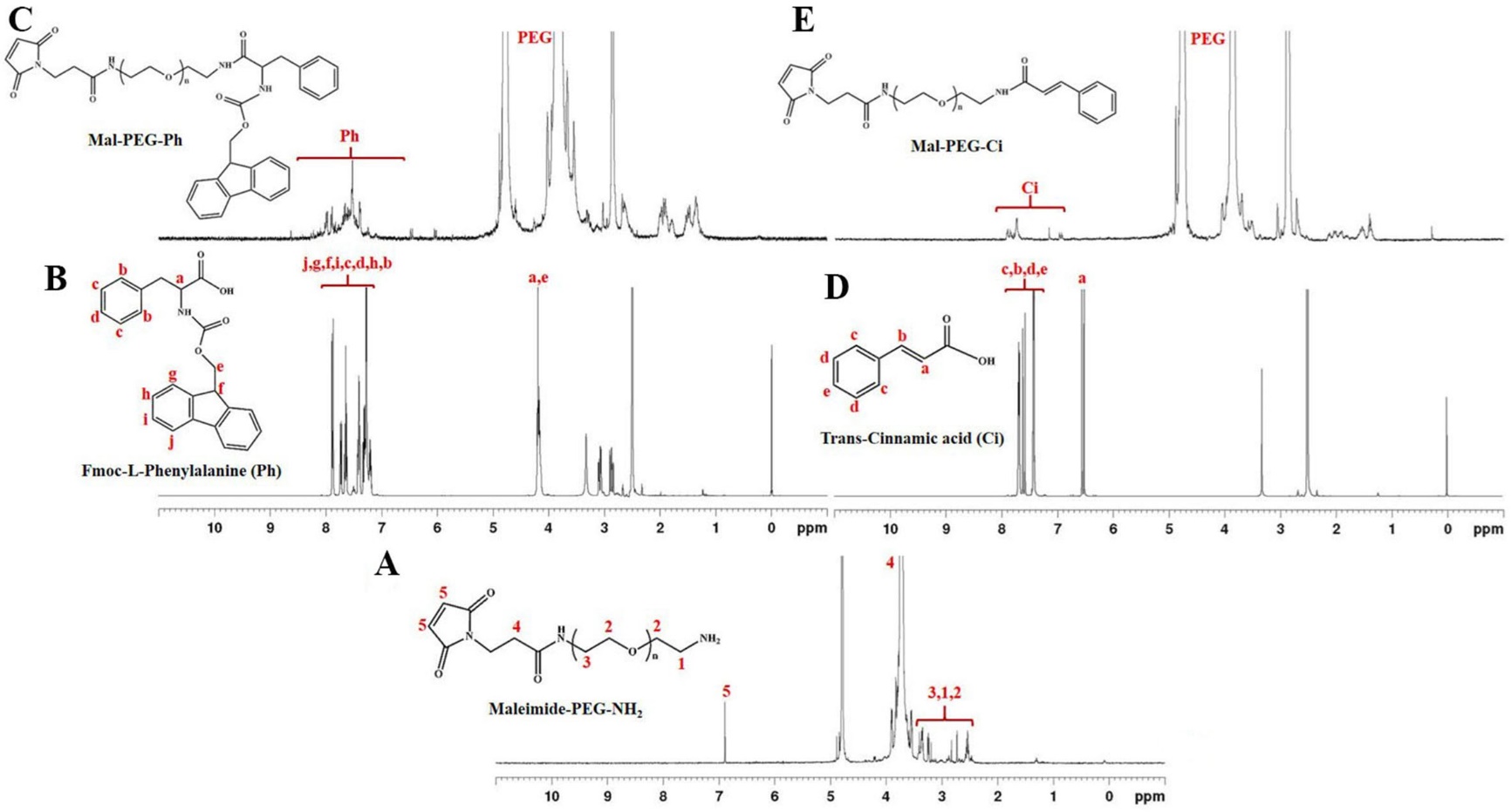


**Fig.S2.** ^1^H-NMR spectra of Maleimide-PEG-NH_2_ (A), Fmoc-L-Phenylalanine (Ph) (B), Mal-PEG-Ph (C), and *Trans*-Cinnamic acid (Ci) (D), and Mal-PEG-Ci (E).


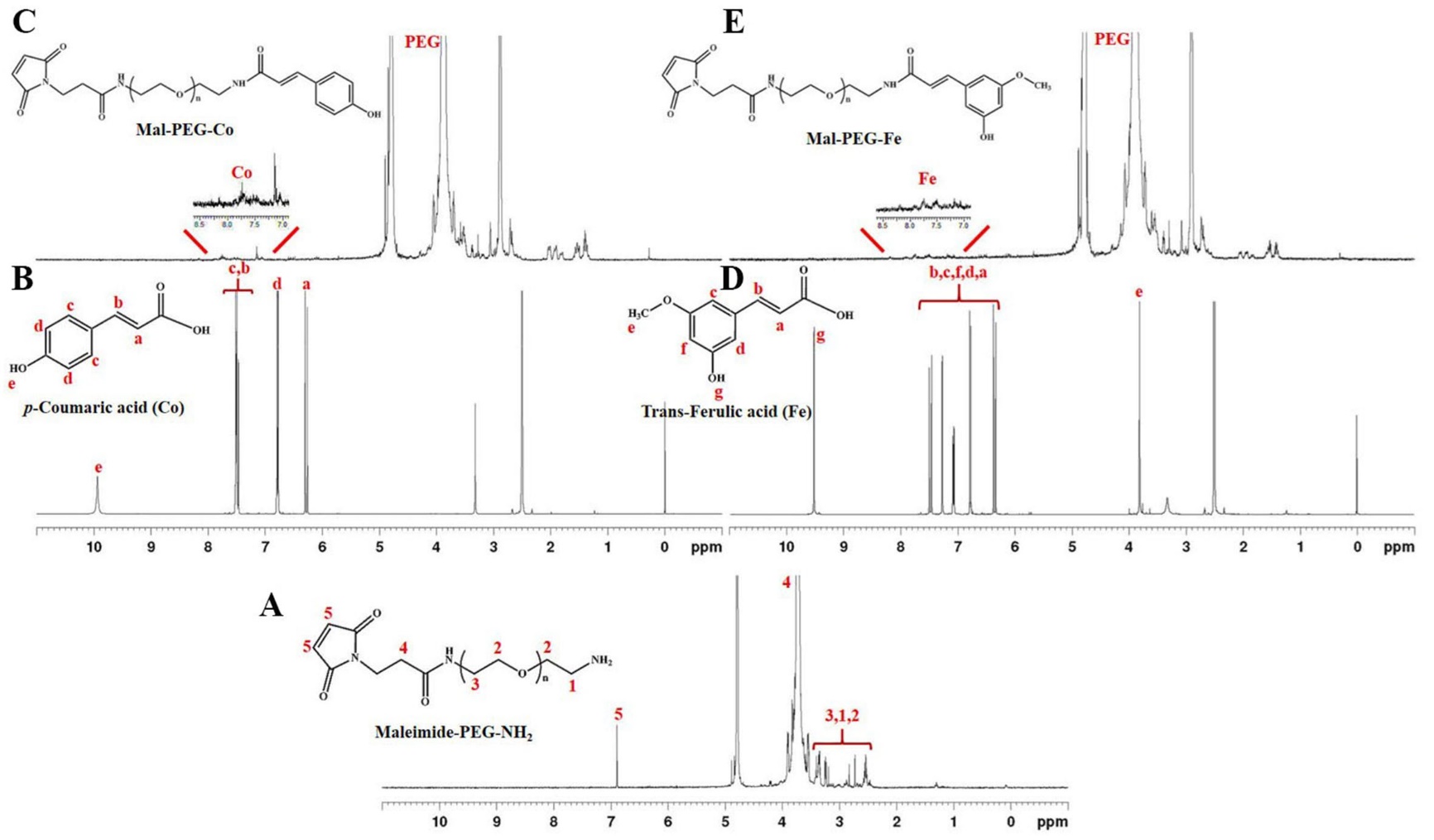


**Fig.S3.** ^1^H-NMR spectra of Maleimide-PEG-NH_2_ (A), *p*-Coumaric acid (Co) (B), Mal-PEG-Co (C), *Trans*-Ferulic acid (Fe) (D), and Mal-PEG-Fe (E).


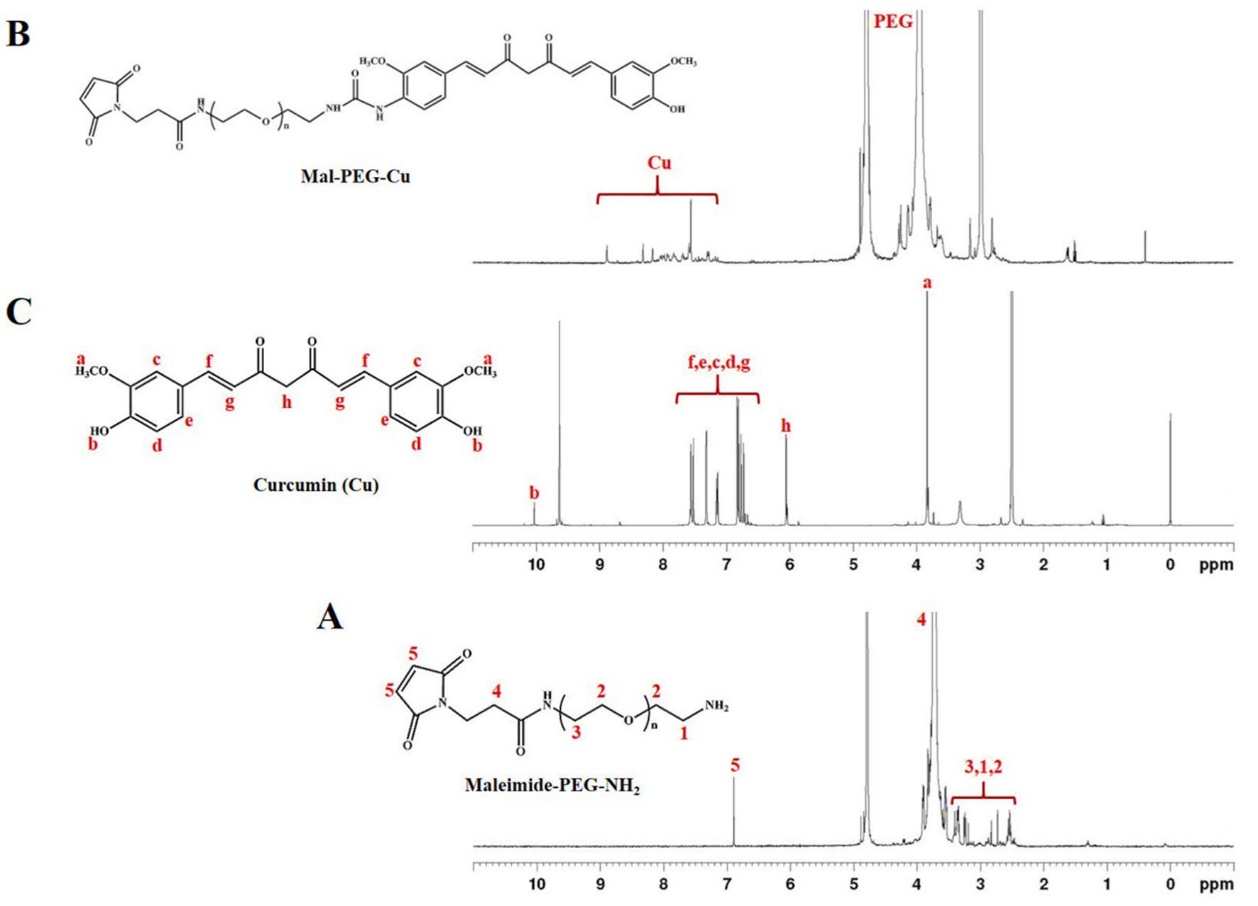


**Fig.S4**. ^1^H-NMR spectra of Maleimide-PEG-NH_2_ (A), Curcumin (Cu) (B), Mal-PEG-Cu (C).


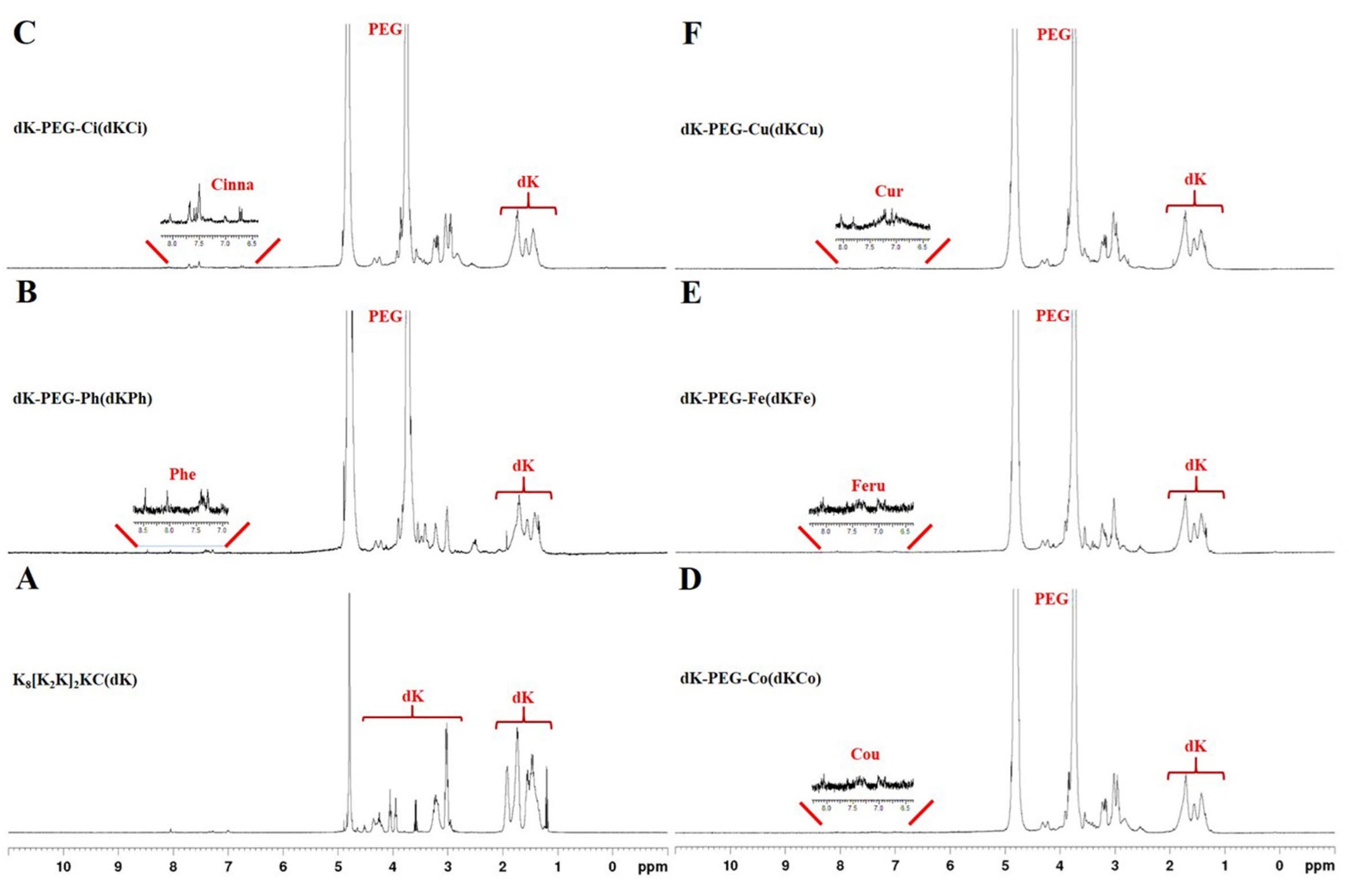


**Fig.S5.** ^1^H-NMR spectra of K_8_[K_2_K]_2_KC (dK) (A), dK-PEG-Ph (dKPh) (B), dK-PEG-Ci (dKCi) (C), dK-PEG-Co (dKCo) (D), dK-PEG-Fe (dKFe) (E), dK-PEG-Cu (dKCu) (F).


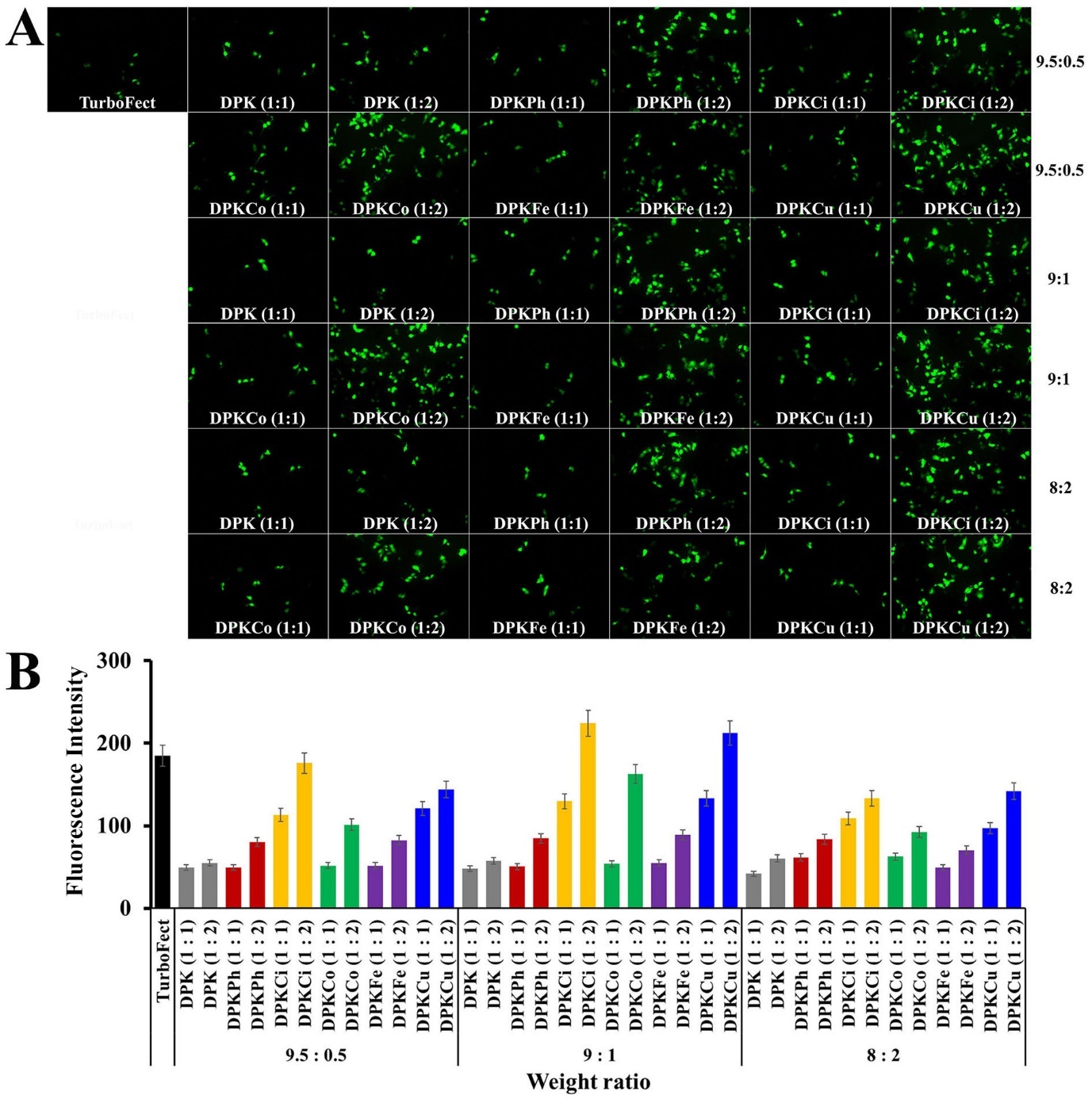


**Fig.S6.** Transfection efficiency of polyplexes in MCF-7 cells. A and B) Polyplexes complexed with pEGFP-N1 at three formulations (5, 10, and 20% dK, without or with targeting ligands) and two w/w ratios of 1:1 and 2:1 were incubated for 48 h in MCF-7 cells. GFP expression was visualized under an inverted fluorescence microscope (A) and evaluated by using a fluorescence spectrometer (B).


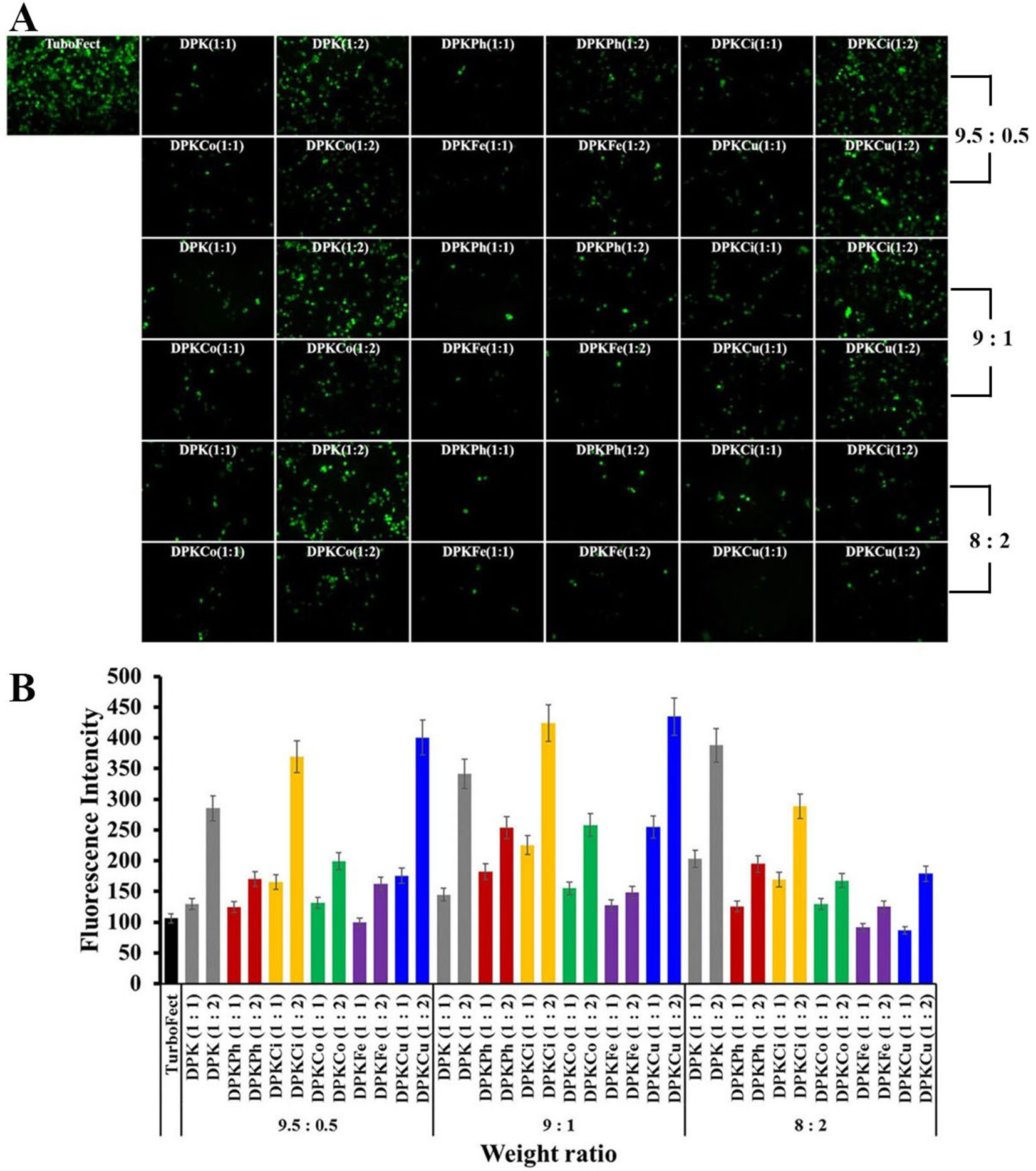


**Fig.S7.** Transfection efficiency of polyplexes in SKBr3 cells. Polyplexes complexed with pEGFP-N1 at three formulations (5, 10, and 20% dK without or with targeting ligands) and two w/w ratios of 1 and 2 was incubated for 48 h in SKBr3 cells. GFP expression was visualized under inverted fluorescence microscopy and evaluated by using fluorescence spectrometer.


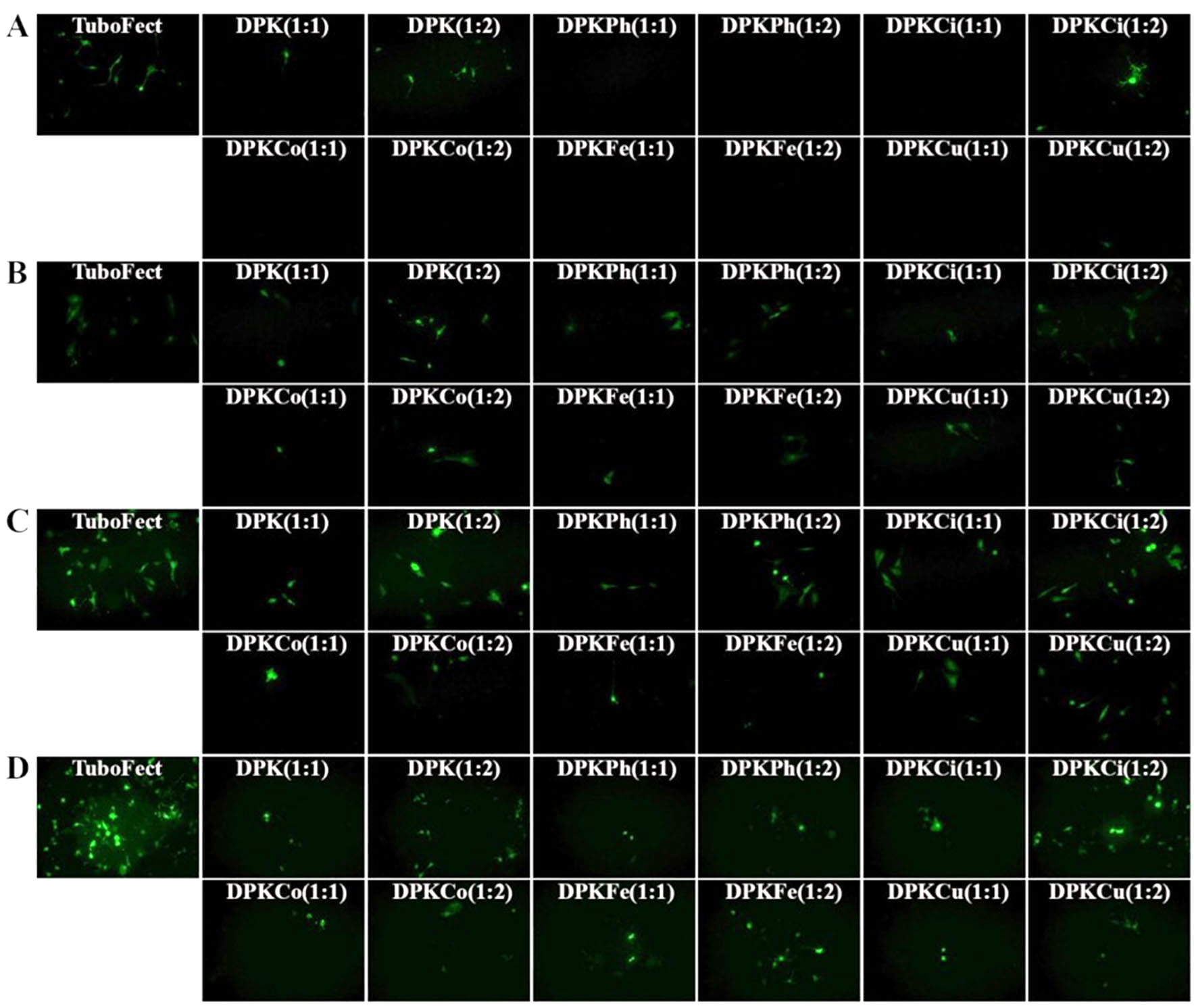


**Fig.S8**. Transfection efficiency of polyplexes in MDA-MB-231 (A), SKOV-3 (B), PC3 (C), and DLD-1. Polyplexes complexed with pEGFP-N1 at 10% dK without or with targeting ligands and two w/w ratios of 1 and 2 was incubated for 48 h in tested cells. GFP expression was visualized under inverted fluorescence microscopy.


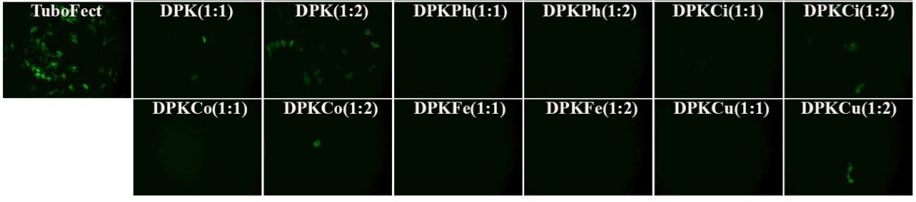


**Fig.S9.** Transfection efficiency of pDNA/copolymer complexes in HaCaT cells. Polyplexes complexed with pEGFP-N1 at 10% dK without or with targeting ligands and two w/w ratios of 1 and 2 was incubated for 48 h in HaCaT cells. GFP expression was visualized under inverted fluorescence microscopy.


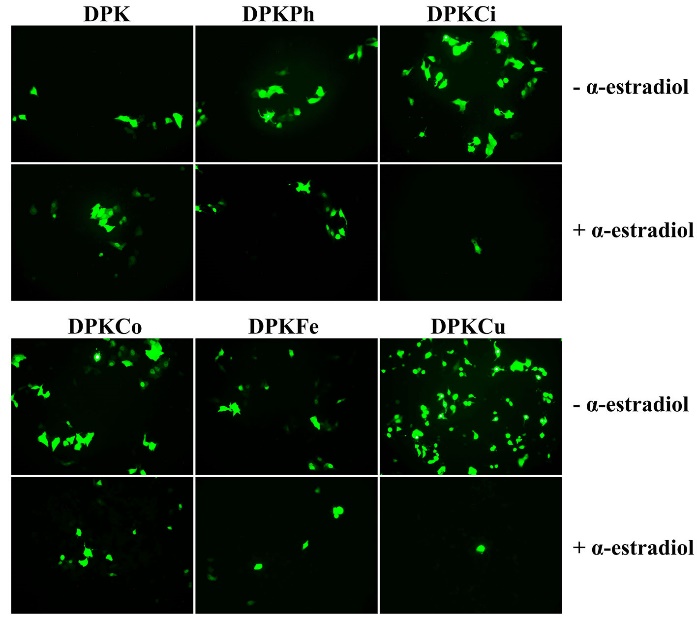


**Fig.S10.** Inhibition of gene transfection via pre-incubation of α-estradiol in MCF-7 cells. After non-treatment or treatment with 1000 ng/mL α-estradiol for 1 h, polyplexes (1:2, w/w) were incubated with pEGFP-N1 for 12 h.


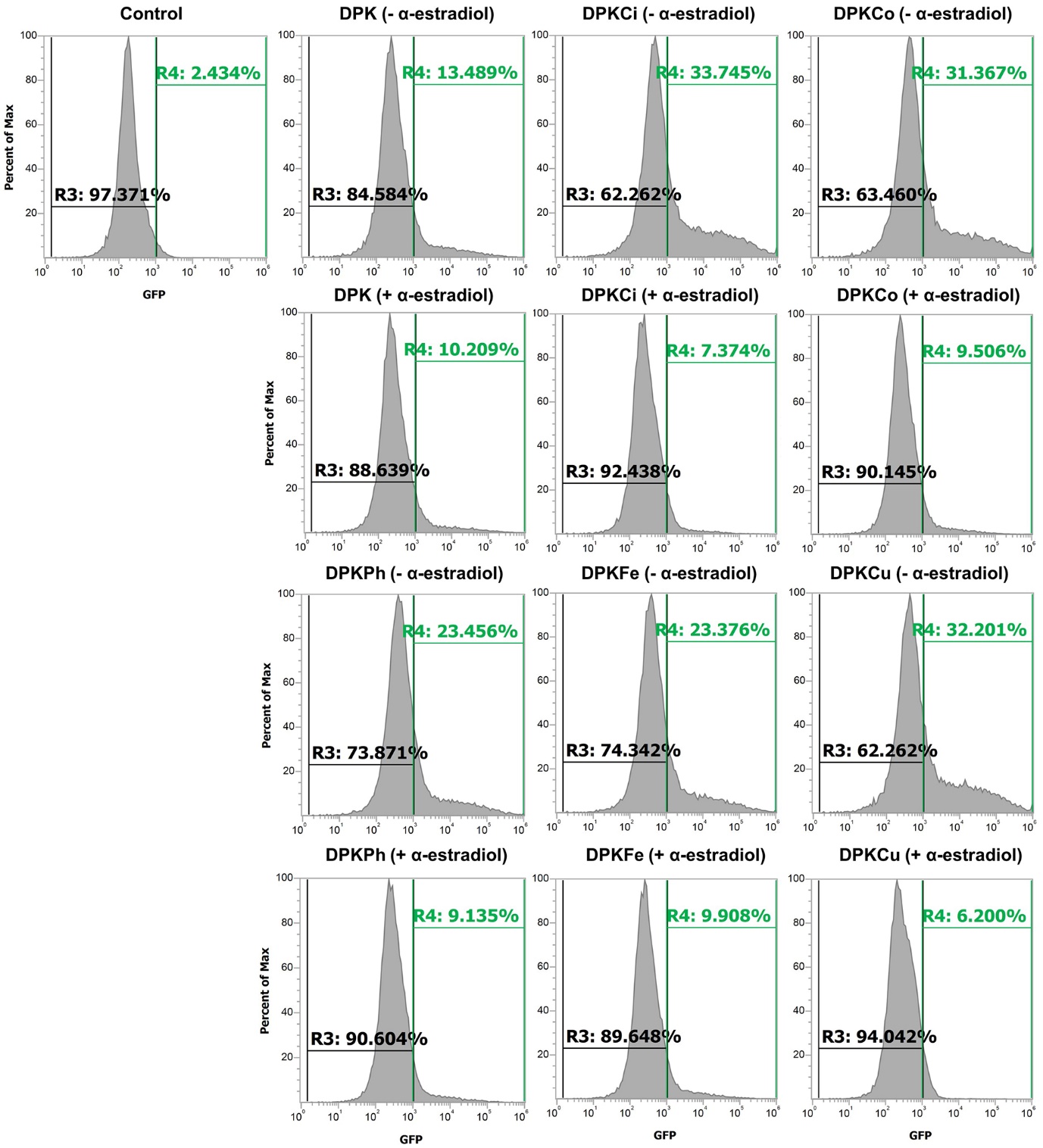


**Fig.S11.** Inhibition of gene transfection via pre-incubation of α-estradiol in MCF-7 cells. After non-treatment or treatment with 1000 ng/mL α-estradiol for 1 h, polyplexes (1:2, w/w) were incubated with pEGFP-N1 for 12 h.
